# Invasion of gut-derived escherichia coli extracellular vesicles exacerbates myocardial ischemia/reperfusion injury

**DOI:** 10.1080/19490976.2026.2635818

**Published:** 2026-02-28

**Authors:** Junzhuo Wang, Ke Hu, He Lu, Ke Chen, Jian Zhang, Shaojun Wu, Lina Kang, Jun Xie, Biao Xu

**Affiliations:** a Jiangsu Key Laboratory for Cardiovascular Information and Health Engineering Medicine, Department of Cardiology, Nanjing Drum Tower Hospital, Affiliated Hospital of Nanjing University Medical School, Nanjing, People's Republic of China; b The Affiliated Drum Tower Hospital of Nanjing Medical University, Nanjing, People's Republic of China; c Nanjing Drum Tower Hospital, Drum Tower Clinical College, Nanjing University of Chinese Medicine, Nanjing, People's Republic of China; d Department of Cardiology, The People's Hospital of Jiawang District of Xuzhou, Xuzhou, People's Republic of China; e Department of Vascular Surgery, Drum Tower Hospital, Affiliated Hospital of Nanjing University Medical School, Nanjing, People's Republic of China; f Department of Cardiology, National Cardiovascular Disease Regional Center for Anhui, the First Affiliated Hospital of Anhui Medical University, Hefei People's Republic of China; g Department of Science and Technology, Nanjing Drum Tower Hospital, Affiliated Hospital of Nanjing University Medical School, Nanjing, People's Republic of China

**Keywords:** Cardiac ischemia-reperfusion injury, Escherichia coli, Extracellular Vesicles, Inflammation

## Abstract

Recent studies have highlighted the close relationship between gut microbiota and the cardiovascular system; however, the precise mechanisms and modes of their interaction remain incompletely understood. Among the various factors involved, bacterial extracellular vesicles (EVs) are often overlooked, despite their potential roles in multiple pathological processes. To investigate the role of bacterial EVs in shaping the inflammatory microenvironment following myocardial ischemia-reperfusion injury, we colonized the intestines of Rosa26.tdTomato reporter mice with *Escherichia coli* (*E. coli*) expressing Cre recombinase. Using FACS-beads and immunofluorescence techniques, we found that myocardial ischemia-reperfusion injury in mice significantly enhanced the invasion of gut-derived bacterial EVs. Meanwhile, in patients with ST-segment elevation myocardial infarction, we also confirmed the invasion of bacterial EVs via the FACS-bead method, and there was a significant correlation between extracellular vesicles in peripheral blood and LPS, suggesting that these EVs can be key carriers for LPS translocation. In this pathological process, invading *E. coli* EVs exacerbate the mobilization and infiltration of systemic and local inflammatory cells, thereby aggravating myocardial damage and impairing cardiac function. Notably, glucagon-like peptide-2 can effectively alleviate inflammatory responses and myocardial injury by inhibiting the translocation of E. coli-derived EVs. In conclusion, our study is the first to confirm the impact of gut-derived EVs on myocardial ischemia-reperfusion injury, revealing that *E. coli* EVs can amplify inflammatory responses. These findings provide new insights into the gut-heart axis and offer a theoretical basis for the therapeutic potential of glucagon-like peptide-2 in cardiovascular diseases.

## Graphical abstract

**Figure:**
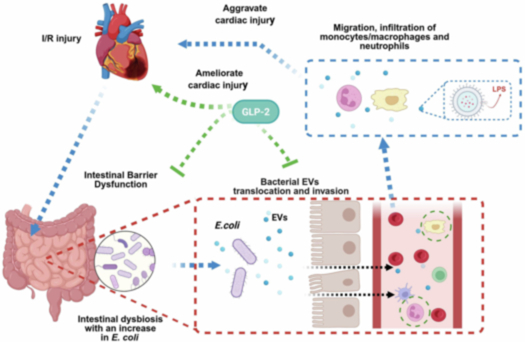

## Introduction

Acute myocardial infarction remains a leading threat to human health. Although advances in medical technology and the widespread adoption of emergency coronary interventions have significantly improved the survival rates and prognoses of acute myocardial infarction patients, ischemia/reperfusion (I/R) injury continues to adversely affect both the survival duration and quality of life in postoperative patients.^1^^,^^2^ Epidemiological studies indicate that even among patients who achieve complete revascularization, the 10-year mortality rate remains alarmingly high.^3^^,^^4^ Emerging evidence suggests that systemic and local mobilization and infiltration of inflammatory cells following acute myocardial injury are closely associated with myocardial damage and repair.^5^ While inflammation is essential for clearing necrotic tissue and promoting subsequent scar formation, excessive inflammatory responses and cell migration can impair cardiac functional recovery and tissue repair. Effective immunomodulation to prevent overactivation of the innate immune response after myocardial infarction has demonstrated significant benefits for cardiac recovery.^6^^,^^7^ Therefore, controlling inflammation following I/R injury is critical to improving cardiac tissue repair and enhancing therapeutic outcomes for myocardial infarction patients.

In recent years, it has become increasingly evident that the gut microbiota plays a pivotal role in maintaining health and influencing disease susceptibility. Dysbiosis has been closely linked to the development and progression of various diseases, including atherosclerosis, hypertension, heart failure, chronic kidney disease, obesity, and type 2 diabetes.^8^ For diseases involving ischemia/reperfusion injury, the role of the gut microbiota has not been fully clarified. Relevant studies have also shown that changes in gut microbiota composition and probiotic supplementation after ischemia/reperfusion injury of the small intestine^9^^,^^10^ and heart^11^ can affect tissue repair and prognosis. Traditionally, the activation of innate immunity after myocardial infarction was primarily attributed to endogenous damage-associated molecular patterns. However, an increasing amount of evidence indicates that in various diseases including myocardial infarction, the levels of pathogen-associated molecular patterns (PAMPs) in the circulatory system and in blood clots, such as lipopolysaccharide (LPS), are elevated.^12-14^ These PAMPs are closely associated with inflammatory responses, disease severity, and patient outcomes. Gut microbiota invasion and alterations in its metabolites have been identified as primary contributors to these phenomena.

In addition to the well-characterized metabolites produced by the gut microbiota, bacteria also secrete extracellular vesicles (EVs). EVs are cell-derived membrane structures that can be divided into two types: exosomes, which are secreted after the fusion of endosomes with the plasma membrane, and microvesicles, which are directly shed from the plasma membrane.^15^ These nanoscale bacterial vesicles can directly participate in various physiological and pathological processes, including autophagy regulation and immune modulation.^16-18^ In cardiovascular diseases, bacterial EVs also have a close association with atherosclerosis.^19^ To date, the role of gut microbiota-derived EVs in myocardial I/R injury remains unexplored, leaving this research area a blank. Therefore, this study aims to investigate the involvement of gut microbiota-derived EVs in the pathophysiological processes of myocardial I/R injury. Specifically, we seek to elucidate their roles in the inflammatory microenvironment following I/R injury and their contributions to the progression of this pathological condition.

Our study demonstrates that bacterial EVs cross the intestinal barrier after myocardial I/R injury, directly influencing both systemic and local inflammatory responses. These findings provide novel therapeutic insights and strategies, highlighting the potential of enhancing intestinal barrier function to promote cardiac repair.

## Materials and methods

A detailed description of the methods can be found in the Supplemental Materials.

### Ethical approval

The research involving human subjects was conducted in accordance with the Declaration of Helsinki and received approval from the Institutional Ethics Committee of the First Affiliated Hospital of Anhui Medical University (Approval No. 20230505). Informed consent was obtained from all participants. All animal experiments were approved by the Ethics Committee of Nanjing Drum Tower Hospital (Approval No. 2023AE01089) and carried out in compliance with the European Parliament Directive 2010/63/EU. At the end of the experiments, all animals were anesthetized with 1.5% to 2% isoflurane and subsequently euthanized by cervical dislocation. Further details are provided in the supplementary materials.

### Statistical analysis

All data were determined from multiple individual biological samples and presented as mean values ± standard error of the mean. The “n” in the study represented the number of biological replicates, which is indicated in the figure legends. GraphPad Prism 9 was used to perform the statistical analysis. The data were first subjected to a normality test, followed by analysis using unpaired Student's t-test or one-way ANOVA combined with Tukey's multiple comparisons test. When comparing multiple groups, we conducted pairwise comparisons between each group. Correlations between continuous variables were determined using Pearson’s correlation or Spearman’s correlation. A *p*-value < 0.05 indicated a statistically significant difference.

## Results

### Gut microbiota alterations following myocardial I/R injury and characterization of *E. coli* EVs

**Figure 1.**
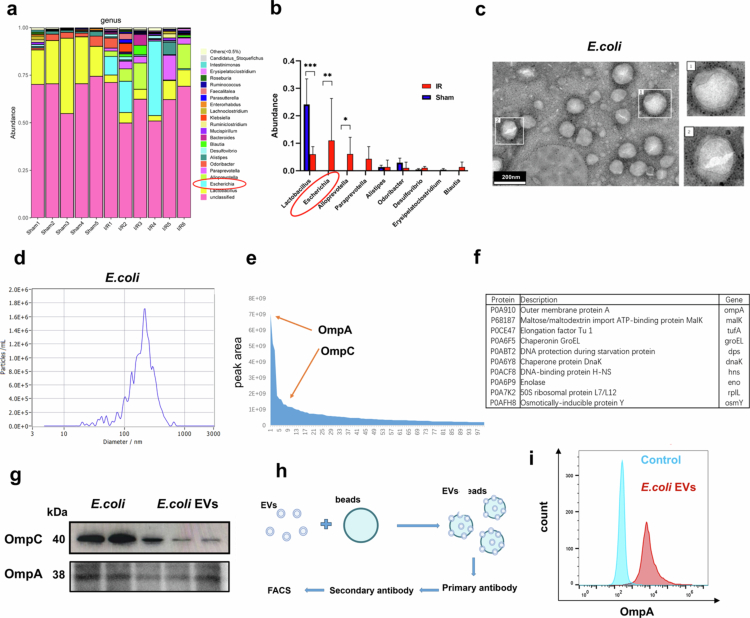
Increased abundance of *E. coli* following I/R injury and characterization of *E. coli* EVs. a. Gut microbiota composition at the genus level in the sham surgery group and the I/R group (3 days after I/R injury). **b.** Comparative analysis of bacterial abundance at the genus level between the sham surgery group and the I/R group. **c.** Transmission electron microscopy images of *E. coli* EVs. Scale bar: 200 nm. **d.** Nanoparticle tracking analysis of *E. coli* EVs. **e.** Proteomic analysis of the dynamic range of proteins in *E. coli* EVs, with relative expression levels of OmpC and OmpA indicated by orange red arrows. **f.** Top 10 most abundant proteins in *E. coli* EVs. **g.** Representative western blot images showing OmpA and OmpC expression. **h.** Schematic diagram of the FACS-beads protocol for analyzing bead-bound EV proteins. **i.** Representative histograms showing OmpA levels in isolated *E. coli* EVs compared to the control.

To investigate the gut microbiota dysbiosis in mice following myocardial I/R injury, we first analyzed the results of 16S ribosomal RNA gene sequencing of colonic contents from mice 3 days after I/R injury modeling surgery, previously conducted by our research group.^20^ We observed significant alterations in the gut microbiota composition post-I/R injury. At the genus level, the proportion of *Escherichia* markedly increased following I/R injury, while the proportion of the recognized probiotic *Lactobacillus* significantly decreased (Figure 1a-b**, Figure S1**). The relatively high proportion of *Escherichia coli* (*E. coli*) and its increased abundance following I/R injury are likely to have a substantial impact on the progression of I/R injury. Therefore, we selected *E. coli* as the primary focus for subsequent research, aiming to investigate the effects of *E. coli* EVs on myocardial injury repair.

We employed ultracentrifugation with a density gradient to isolate *E. coli* EVs and subsequently characterized their phenotypic and morphological features. Transmission electron microscopy imaging revealed that the EVs exhibit a bilayer membrane structure (Figure 1c). Nanoparticle tracking analysis indicated that the *E. coli* EVs had an average particle size of approximately 200 nm (Figure 1d). Proteomic analysis of the isolated *E. coli* EVs demonstrated abundant expression of OmpC and OmpA, both of which are major components of the bacterial outer membrane (Figure 1e-f**, Table S1**). Western blot analysis further confirmed the presence of OmpC and OmpA in the *E. coli* EVs (Figure 1g). Finally, using a flow cytometry-based protocol to label proteins on bead-bound EVs (FACS-beads),^21^ we validated the surface expression of OmpA on *E. coli* EVs (Figure 1h-i). As a negative control when required, we cultured the well-established probiotic *Lactobacillus plantarum* (*L. plantarum*), isolated its EVs, and further characterized them through electron microscopy, particle size analysis, and proteomic profiling (**Figure S2, Table S2**).

In summary, 16S ribosomal RNA gene sequencing of colonic contents from mice with myocardial I/R injury revealed a significant increase in the abundance of *Escherichia coli*. Furthermore, we successfully isolated and characterized *E. coli* EVs.

### Intestinal barrier disruption and translocation of *E. coli* EVs following myocardial I/R injury

**Figure 2.**
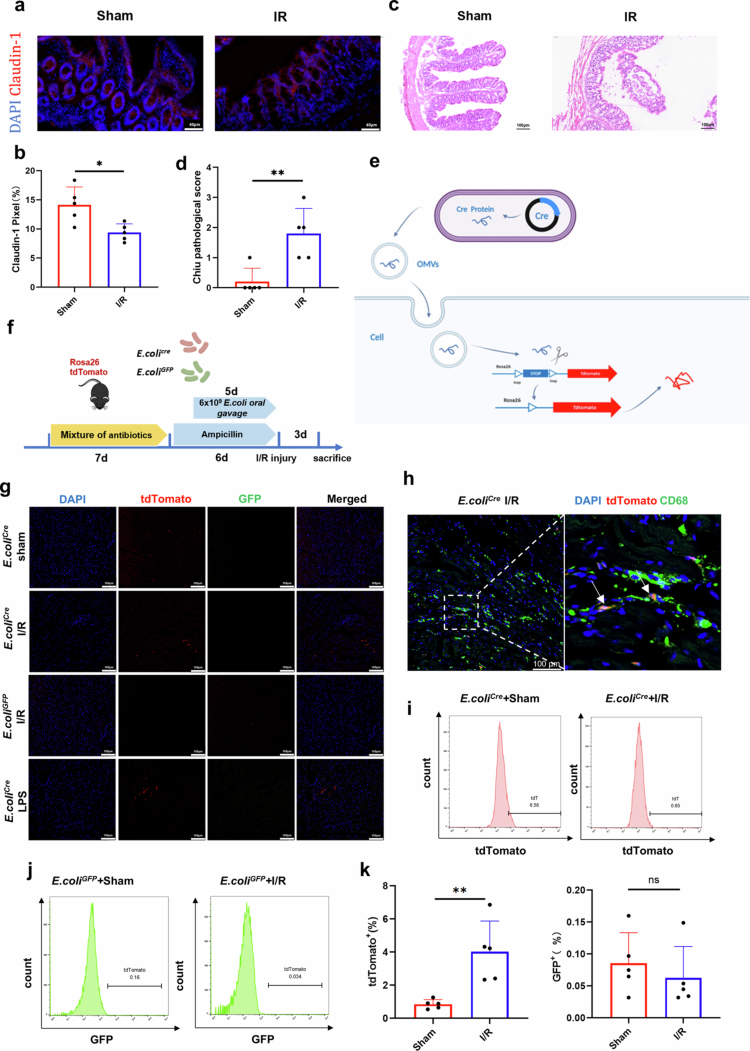
Gut microbiota dysbiosis and translocation of *E. coli* EVs following myocardial I/R injury. a-b. Representative immunofluorescence images of Claudin-1 (red) and DAPI (blue) in ventricular tissues from mice subjected to myocardial I/R injury for 3 days, along with quantitative results (*n* = 5 mice). Scale bar: 40 μm. **c-d.** Representative HE staining images of intestinal tissue from mice subjected to myocardial I/R injury for 3 days, along with modified Chiu's score quantitative results (*n* = 5 mice). Scale bar: 100μm. **e.** Schematic diagram of the in vivo experimental procedure. **f.** Schematic diagram of *E. coli*^Cre^ in combination with Rosa26.tdTomato reporter mice to visualize the transfer of *E. coli* EVs. **g.** Representative immunofluorescence images of ventricular tdTomato (red), GFP (green), and DAPI (blue) staining in cardiac tissues of Rosa26.tdTomato reporter mice colonized with *E. coli*^Cre^ or *E. coli*^GFP^ following 3 days of myocardial I/R injury, along with quantitative results (*n* = 5). Scale bar: 100μm. **h.** Representative immunofluorescence images of ventricular tdTomato (red), CD68 (green), and DAPI (blue) staining in cardiac tissues of Rosa26.tdTomato reporter mice colonized with *E. coli*^Cre^ following 3 days of myocardial I/R injury. The arrow indicates co-staining of CD68 and tdTomato. Scale bar: 100μm. **i-k.** Representative histograms showing tdTomato and GFP expression in peripheral blood in Rosa26.tdTomato reporter mice colonized with *E. coli*^Cre^ or *E. coli*^GFP^ following 3 days of myocardial I/R injury, along with statistical results (*n* = 5 mice). (ns, not significant, *P < 0.05, **P < 0.01, ***P < 0.001).

We hypothesized that *E. coli* EVs can cross the intestinal mucosal barrier and enter the bloodstream. Therefore, understanding changes in the gut barrier following myocardial I/R injury is crucial for our subsequent studies. To investigate this, we examined whether the intestinal mucosal barrier was altered in mice after I/R injury modeling. On day 3 post-I/R surgery, immunofluorescence staining and Western blot analysis were performed on the intestine. The results showed that compared with the sham operation group, the expressions of Claudin-1 and Occludin were significantly decreased (Figure 2a-b**, Figure S3a**), and these proteins are key tight junction proteins that maintain the intestinal epithelial barrier. We also administered FITC-dextran to mice via intragastric injection, and found that the fluorescence intensity in the serum of mice after myocardial ischemia/reperfusion surgery was significantly increased (**Figure S3b**). Additionally, mechanical barrier damage was observed, as indicated by a marked increase in the modified Chiu's Score^22^ (Figure 2c-d). These findings further demonstrate the disruption of the intestinal mucosal barrier. Consistent with previous studies,^12^^,^^20^ our results suggest that myocardial I/R injury-induced ischemia can lead to intestinal barrier damage.

To determine whether bacterial extracellular vesicles can cross the intestinal mucosal barrier, we referenced the study by Miriam Bittel and colleagues^23^ and utilized *Escherichia coli* engineered to express Cre recombinase (*E. coli*^Cre^) in combination with Rosa26.tdTomato reporter mice to visualize the transfer of *E. coli* EVs to peripheral blood and cardiac tissues. RT-PCR experiments confirmed the presence of Cre expression in *E. coli*^Cre^ EVs (**Figure S4a**). When these EVs enter mouse cells, the Cre-LoxP system induces the expression of tdTomato red fluorescence in the recipient cells (Figure 2e). We first used a mixture of antibiotics to clear the endogenous gut microbiota in Rosa26.tdTomato reporter mice and then colonized the gut with *E. coli*^Cre^ via oral gavage. To validate the efficacy of microbial depletion, we performed quantitative analysis of bacterial load in collected fecal samples (**Figure S4b**). Engineered Escherichia coli expressing green fluorescent protein (GFP) (*E. coli*^GFP^) was used as a control (Figure 2f). After colonization with *E. coli*^Cre^, mice underwent I/R surgery, and tdTomato red fluorescence was detected in cardiac tissues (Figure 2g), consistent with results observed in mice with intestinal barrier injury induced by LPS intraperitoneal injection. In contrast, no GFP fluorescence was detected in mice colonized with *E. coli*^GFP^, indicating that the tdTomato red fluorescence resulted from the translocation of *E. coli*^Cre^ EVs, not from the translocation of *E. coli*^Cre^ itself. Considering that EVs can be phagocytosed by macrophages, we performed CD68 staining in cardiac tissues and indeed observed co-localization signals of CD68 and tdTomato (Figure 2h). Flow cytometry analysis of peripheral blood further supported these findings, showing an increased number of cells expressing tdTomato red fluorescence in the blood after I/R injury (Figure 2i-k). These results demonstrate that following myocardial I/R injury, more bacterial EVs enter the peripheral circulation and cardiac tissues.

### Bacterial EVs-mediated LPS translocation in myocardial I/R injury

In addition to membrane protein markers such as OmpC and OmpA, *E. coli* derived EVs also contain pathogenic factors including LPS, as well as LPS assembly-related proteins like LptD and LptE, which are absent in the control *L. plantarum* EVs (**Table S1-S2**). While numerous studies have demonstrated that elevated circulating LPS levels in myocardial infarction patients primarily result from gut bacterial translocation, our findings reveal that bacterial EV translocation also serves as a critical contributor to increased bacterial load in the bloodstream. This indicates that changes in biomarkers such as LPS cannot be solely attributed to the translocation of intact bacteria.

**Figure 3.**
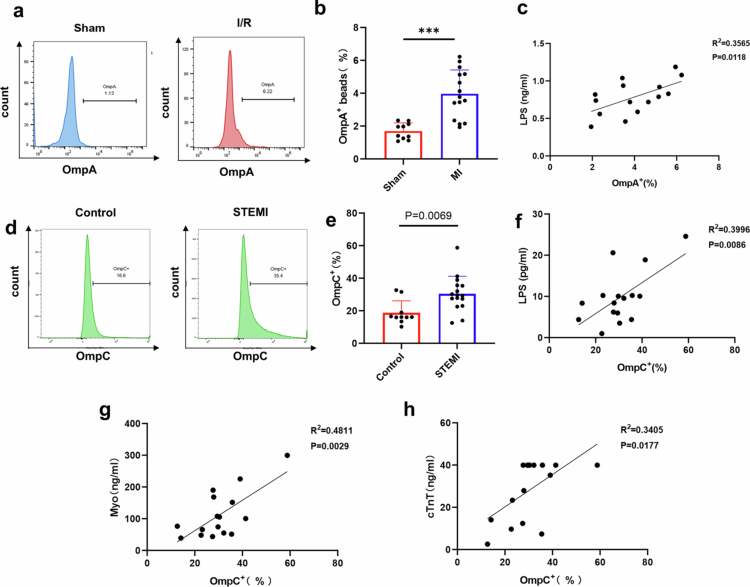
Bacterial extracellular vesicles mediate LPS translocation in cardiac injury. a-b. Representative histograms showing the levels of OmpA in EVs isolated from the peripheral blood of mice with myocardial I/R injury for 1 day, compared with the control group, along with statistical results (*n* = 5 mice). **c.** Correlation between serum OmpA^+^ beads and LPS in the peripheral blood of mice with myocardial I/R injury for 1 days. **d-e.** Representative histograms showing the levels of OmpC in EVs isolated from peripheral blood in healthy controls (*n* = 10 donors) and STEMI patients (*n* = 16 donors), along with statistical analysis. **f-g.** Correlation between serum OmpC^+^ beads and LPS, myoglobin (Myo), high-sensitivity cardiac troponin T (cTnT) in the peripheral blood of patients with STEMI (*n* = 16 donors). (ns, not significant, *P < 0.05, **P < 0.01, ***P < 0.001).

To further validate this finding, we collected peripheral blood samples one day after establishing the mouse I/R model. EVs were isolated by ultracentrifugation and analyzed using FACS-beads assays for OmpA expression. Consistent with the elevated levels of *E. coli* derived EVs in circulation after myocardial I/R injury (Figure 3a-b), we identified a significant correlation between OmpA^+^ EVs and circulating LPS concentrations (Figure 3c). Beyond murine studies, we recruited both healthy controls and ST-segment elevation myocardial infarction (STEMI) patients (**Table S3**), collecting peripheral blood samples during the acute phase of myocardial infarction. Given that human peripheral blood volume is significantly greater than that of mice, resulting in substantially higher EV yields, the OmpA positivity rate detected by FACS-beads assays was excessively high. Therefore, we alternatively examined OmpC, another marker protein of EVs, in human peripheral blood samples. Our results demonstrated significantly elevated levels of OmpC^+^ EVs in STEMI patients compared to healthy controls (Figure 3d-e). Importantly, consistent with our animal studies, we observed a strong correlation between the abundance of bacterial EVs and circulating LPS levels in human peripheral blood (Figure 3f), suggesting that bacterial EVs serve as vehicles for LPS translocation into the bloodstream during STEMI. Furthermore, we found that the proportion of OmpC^+^ EVs showed significant positive correlations with established myocardial injury markers, including myoglobin (Myo) and high-sensitivity cardiac troponin T (cTnT) (Figure 3g-h). These findings collectively indicate that the extent of myocardial ischemic necrosis directly influences the degree of LPS-containing bacterial EV translocation into circulation.

This study provides compelling evidence that bacterial EVs can translocate independently of intact bacteria and identifies them as important carriers for systemic LPS dissemination, thereby contributing to the inflammatory burden. Our findings highlight the potential therapeutic value of targeting gut-derived bacterial EVs to attenuate PAMPs-mediated damage, offering a novel strategic approach for myocardial infarction treatment.

### Impact of *E. coli* EVs on monocytes/macrophages recruitment and polarization

**Figure 4.**
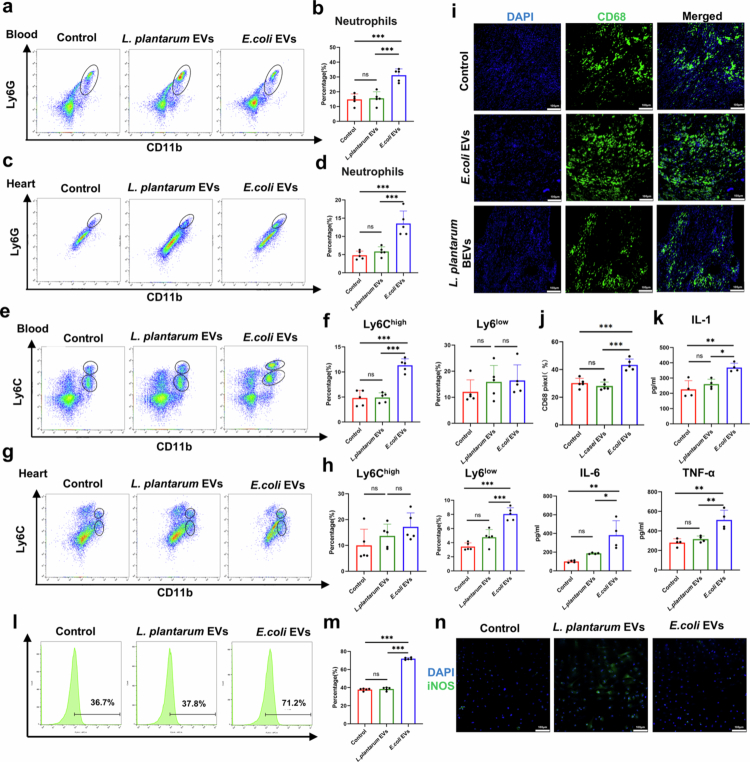
*E. coli* EVs promote systemic and cardiac inflammation and pro-inflammatory monocytes/macrophages polarization. a-b. Representative flow cytometry plots showing neutrophils (CD45^+^CD11b^+^Ly6G^+^) in peripheral blood of each group at 3 days after oral gavage and myocardial I/R injury, along with quantitative analysis (*n* = 5 mice). **c-d.** Representative flow cytometry plots showing neutrophils (CD45^+^CD11b^+^Ly6G^+^) in cardiac tissue of each group at 3 days after oral gavage and myocardial I/R injury, along with quantitative analysis (*n* = 5 mice). **e-f.** Representative flow cytometry plots showing Ly6C^high^ monocytes and Ly6C^low^ monocytes/macrophages (CD45^+^CD11b^+^) in peripheral blood of each group at 3 days after oral gavage and myocardial I/R injury, along with quantitative analysis (*n* = 5 mice). **g-h.** Representative flow cytometry plots showing Ly6C^high^ monocytes and Ly6C^low^ monocytes/macrophages (CD45^+^CD11b^+^) in cardiac tissue of each group at 3 days after oral gavage and myocardial I/R injury, along with quantitative analysis (*n* = 5 mice). **i-j.** Representative immunofluorescence images of ventricular tissue stained for CD68 (green) and DAPI (blue) of each group at 3 days after oral gavage and myocardial I/R injury, along with quantitative analysis (*n* = 5 mice). Scale bar: 100 µm. **k.** Statistical results of peripheral blood inflammatory cytokines (IL-1β, IL-6, and TNF-*α*) of each group detected by ELISA (*n* = 5 mice). **l-m.** Representative histograms and statistical results of Ly6C expression in RAW264.7 cells treated with *E. coli* EVs and *L. plantarum* EVs for 24 hours, as detected by flow cytometry (*n* = 5 mice). **n.** Representative immunofluorescence images of iNOS (green) and DAPI (blue) staining in RAW264.7 cells treated with *E. coli* EVs or *L. plantarum* EVs for 24 hours, along with quantitative analysis (*n* = 5 mice). Scale bar: 100 µm. (ns, not significant, *P < 0.05, **P < 0.01, ***P < 0.001).

Given that *E. coli* EVs contain PAMPs such as LPS, the entry of *E. coli* EVs into circulation and cardiac tissue is expected to influence both systemic and local inflammatory responses. To investigate this, we first performed flow cytometry analysis to evaluate the number of neutrophils in the peripheral circulation and cardiac tissue of mixture antibiotic-treated (ABX) mice. These mice underwent myocardial infarction followed by I/R injury, and were then treated via oral gavage with *E. coli* EVs or *L. plantarum* EVs. Three days after IR injury, we observed a significant increase in neutrophils in the *E. coli* EV-treated group compared to the control and *L. plantarum* EV-treated groups (Figure 4a-d; The specific gating strategy is shown in **Figure S5**). In terms of monocytes/macrophages, *E. coli* EVs notably promoted the pro-inflammatory Ly6C^high^ phenotype, indicating increased pro-inflammatory polarization of monocytes/macrophages in peripheral blood and cardiac tissue (Figure 4e-h). Immunofluorescence staining of cardiac tissue for CD68, a macrophage marker (Figure 4i-j), HE staining showing inflammatory cell infiltration^24^ (**Figure S6**), and ELISA assays measuring IL-1β, IL-6, and TNF-*α* levels in peripheral blood (Figure 4k) further confirmed that *E. coli* EVs crossing the intestinal mucosal barrier significantly promote both systemic and local cardiac inflammation, whereas *L. plantarum* EVs exhibited no such effect.

Given that the spleen serves as a reservoir for monocytes and myocardial I/R injury can mobilize splenic macrophages, we also assessed changes in monocytes/macrophages within the spleen. We found that after oral gavage with *E. coli* EVs, the number of monocytes/macrophages in the spleen decreased, while macrophage mobilization significantly increased (**Figure S7a-b**). Subsequently, we conducted in vitro experiments using the RAW264.7 cell line as a model. Cells were treated with either *E. coli* EVs or *L. plantarum* EVs. The results showed that *E. coli* EVs significantly influenced the differentiation of RAW264.7 cells in a dose-dependent manner (Figure 4l-m**, Figure S7c-d**). Immunofluorescence staining for iNOS further confirmed that *E. coli* EVs promoted monocytes/macrophages polarization toward a pro-inflammatory phenotype (Figure 4n). To further clarify whether the promotion of inflammatory responses is associated with LPS, we conducted additional in vitro experiments using EVs derived from *Lactobacillus casei* and *Bacteroides fragilis*. We found that the results were consistent with our previous findings: EVs isolated from *Bacteroides fragilis* (a bacterium containing LPS) exhibited a more pronounced pro-inflammatory effect (**Figure S8a-b**). Numerous studies have confirmed that LPS can regulate systemic inflammatory responses through Toll-like receptor 4 (TLR4). We attempted to verify the effect of E. coli EVs on TLR4 using HEK-Blue™ mTLR4 cells.^25^ Our results showed that E. coli EVs could effectively activate the TLR4 signaling pathway (**Figure S8c**). Moreover, similar to their effect on macrophage polarization, the activation intensity of the TLR4 signaling pathway increased with the increase in EVs concentration.

In summary, these findings demonstrate that *E. coli* EVs crossing the intestinal mucosal barrier promote the recruitment of inflammatory cells to systemic and cardiac tissues, enhance the mobilization of splenic monocytes/macrophages, and drive the pro-inflammatory polarization of monocytes/macrophages.

### *E. coli* EVs exacerbate cardiac dysfunction and fibrosis post-I/R injury

**Figure 5.**
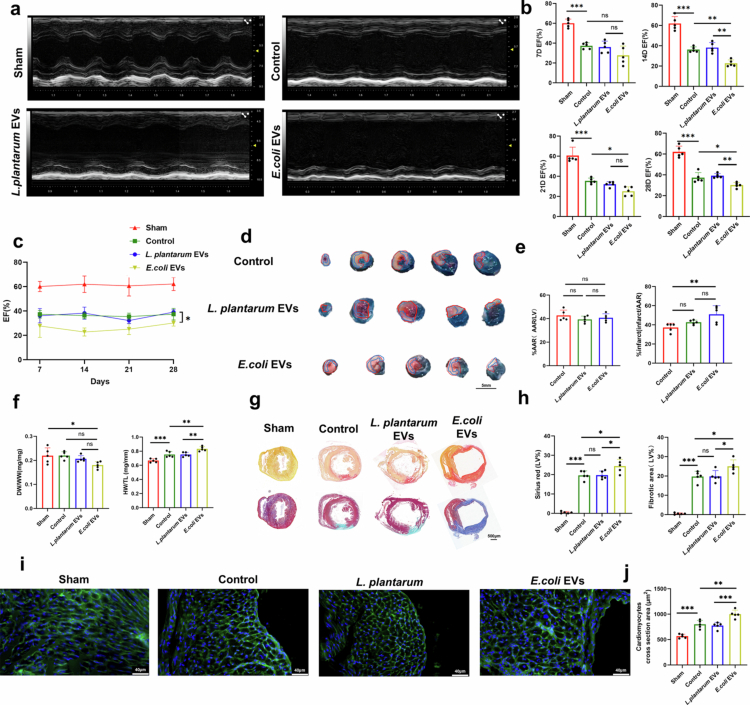
*E. coli* EVs Exacerbates Cardiac Dysfunction. a. Left ventricular function assessed using animal echocardiography (representative long-axis parasternal M-mode echocardiography). **b-c.** Statistical results of left ventricular ejection fraction (EF) at different time points (*n* = 5 mice). **d.** Representative images of Evans Blue/TTC staining showing myocardial ischemia-reperfusion injury 3 days post-operation in mice. Area-at-risk (AAR) is outlined within the red line, and infarct area is outlined within the blue line. Scale bar: 5 mm. **e.** Quantitative analysis of the AAR/left ventricle ratio and infarct area/AAR ratio (*n* = 5 mice). **f.** Heart weight-to-tibia length ratio (HW/TL) at 4 weeks post-I/R surgery and lung wet-to-dry weight ratio (WW/DW) at 3 days post-surgery in mice (*n* = 5 mice). **g-h.** Representative images of Masson's trichrome staining and Sirius Red staining of mouse hearts at 28 days post- myocardial I/R injury, along with quantitative analysis (*n* = 5 mice). Scale bar: 500 µm. **i-j**. Representative Wheat Germ Agglutinin staining images of the peri-infarct region in mouse myocardium 28 days post-I/R surgery, along with quantitative analysis of cardiomyocyte size (*n* = 5 mice). Scale bar: 40 µm. (ns, not significant, *P < 0.05, **P < 0.01, ***P < 0.001).

The findings demonstrate that *E. coli* EVs significantly exacerbate the inflammatory response following myocardial I/R injury. Excessive inflammation adversely affects cardiac function, fibrosis, and prognosis in myocardial infarction patients. To further investigate whether *E. coli* EVs crossing the intestinal mucosal barrier directly contribute to more severe cardiac damage, we analyzed their effects in ABX-treated mice subjected to oral gavage with *E. coli* EVs. Following I/R surgery, cardiac function in the *E. coli* EV-treated group showed significant impairment compared to controls (Figure 5a-c). This impairment was attributed to heightened inflammatory infiltration during the early injury phase, which led to persistently reduced cardiac function during the recovery phase, particularly 2–4 weeks post-surgery, compared to the control and *L. plantarum* EV-treated groups.

Evans Blue and 2, 3, 5-triphenyltetrazolium chloride (TTC) staining revealed that the infarct area was significantly larger in the *E. coli* EV-treated group compared to the untreated group, despite similar ischemic areas (Figure 5d-e). Additionally, differences in the lung wet-to-dry weight ratio (WW/DW) and heart weight-to-tibia length ratio (HW/TL) indicated greater cardiac hypertrophy and more severe pulmonary edema in the *E. coli* EV-treated group (Figure 5f). Wheat Germ Agglutinin staining further confirmed myocardial cell enlargement (Figure 5i-j).

Masson’s trichrome and Sirius Red staining of cardiac tissue revealed more pronounced fibrosis and collagen deposition in the *E. coli* EV-treated group (Figure 5g-h), correlating with the observed deterioration in cardiac function. These findings collectively suggest that *E. coli* EVs promote systemic and local inflammatory cell mobilization and infiltration, leading to impaired cardiac function and exacerbated fibrosis. The presence of *E. coli* EVs is closely associated with the severity of cardiac damage.

### GLP-2-mediated inhibition of EV translocation alleviates inflammation and improves cardiac repair

Our findings demonstrate that the gut microbiota composition undergoes significant alterations following cardiac injury, characterized by an increased proportion of *E. coli*. Notably, *E. coli* EVs can breach the intestinal mucosal barrier and influence the inflammatory response during the cardiac repair process. While previous research on gut microbiota has largely focused on the microbes themselves and their metabolites, bacterial EVs have received comparatively little attention as potential therapeutic targets. As a crucial component of the gut-heart-microbiota-immune axis, *E. coli* EVs play a pivotal role in disease progression and recovery. However, developing effective interventions targeting *E. coli* EVs remains a significant challenge for clinical translation.

**Figure 6.**
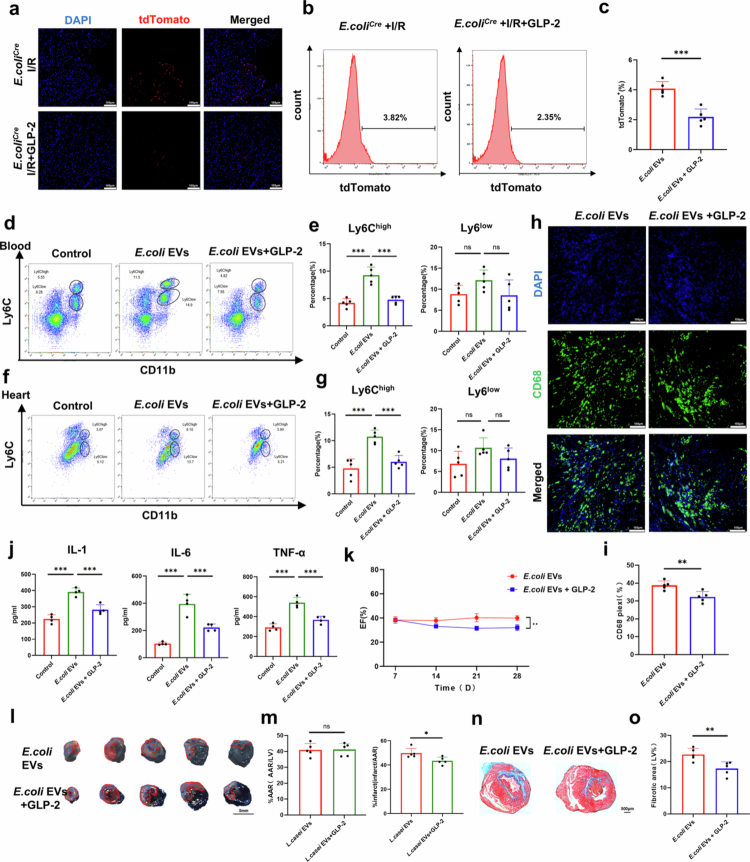
GLP-2's role in inhibiting bacteria EVs translocation and improving cardiac function. a. Representative immunofluorescence images of ventricular tdTomato (red), and DAPI (blue) staining in Rosa26.tdTomato reporter mice colonized with *E. coli*^Cre^ following myocardial I/R injury. In the *E. coli*^Cre^ + GLP-2 group, a degradation-resistant GLP-2 analog (600 µg/kg) was administered subcutaneously 3 days post-I/R surgery. Scale bar: 100µm. **b-c.** Representative histograms showing tdTomato expression in peripheral blood of Rosa26.tdTomato reporter mice colonized with *E. coli*^Cre^, with or without GLP-2 intervention, along with statistical results (*n* = 5 mice). **d-e.** Representative flow cytometry plots showing Ly6C^high^ monocytes and Ly6C^low^ monocytes/macrophages (CD45^+^CD11b^+^) in peripheral blood at 3 days after I/R injury, with or without GLP-2 intervention, along with quantitative analysis (*n* = 5 mice). **f-g.** Representative flow cytometry plots showing Ly6C^high^ monocytes and Ly6C^low^ monocytes/macrophages (CD45^+^CD11b^+^) in cardiac tissue at 3 days after I/R injury, with or without GLP-2 intervention, along with quantitative analysis (*n* = 5 mice). **h-i.** Representative immunofluorescence images of ventricular tissue stained for CD68 (green) and DAPI (blue) 3 days after myocardial I/R injury in mice, with or without GLP-2 intervention, along with quantitative analysis (*n* = 5 mice). Scale bar: 100 µm. **j.** ELISA analysis of peripheral blood inflammatory cytokines (IL-1β, IL-6, and TNF-*α*) in GLP-2 intervention and control groups, along with statistical results. (*n* = 5 mice). **k.** Statistical results of left ventricular ejection fraction (EF) at different time points, with or without GLP-2 intervention (*n* = 5 mice). **l-m.** Representative images of Evans Blue/TTC staining showing myocardial I/R injury 3 days post-operation in mice, with or without GLP-2 intervention. Area-at-risk (AAR) is outlined within the red line, and infarct area is outlined within the blue line, along with quantitative analysis (*n* = 5 mice). Scale bar: 5 mm. **n-o.** Representative images of Masson's trichrome staining of mouse hearts at 28 days post-myocardial I/R injury, with or without GLP-2 intervention, along with quantitative analysis (*n* = 5 mice). Scale bar: 500 µm. (ns, not significant, *P < 0.05, **P < 0.01, ***P < 0.001).

In this context, we have shifted our focus to glucagon-like peptide 2 (GLP-2), a 33-amino acid peptide co-expressed with GLP-1 in the enteroendocrine L-cells of the distal small intestine and colonic mucosa. Emerging evidence indicates that GLP-2 enhances intestinal barrier integrity and provides mucosal protection. Recognizing the challenges of directly targeting bacterial EVs, we aimed to investigate whether GLP-2 could mitigate the translocation of *E. coli* EVs by strengthening the intestinal barrier following myocardial I/R injury, thereby alleviating their detrimental effects on cardiac repair. GLP-1 and GLP-2 may have direct effects on vascular function. To rule out this potential interference, we established a cardiac I/R model in ABX-treated mice and administered GLP-2 treatment. The results showed that in ABX-treated mice, GLP-2 did not directly affect cardiac function or cardiac angiogenesis after cardiac injury (**Figure S9**). Next, we colonized *E. coli*^Cre^ in the intestines of Rosa26.tdTomato reporter mice and established an I/R injury model. Immediately after I/R injury, 600 µg/kg of a degradation-resistant GLP-2 analog was administered subcutaneously, followed by consecutive dosing for three days. The dosing regimen was based on previous relevant studies.^20^^,^^26^^,^^27^ As hypothesized, fluorescence staining of cardiac tissue (Figure 6a) and flow cytometry analysis of peripheral cells (Figure 6b-c) revealed a significant reduction in tdTomato^+^ cells, indicating that the translocation of *E. coli* EVs was effectively suppressed. Furthermore, we confirmed that GLP-2 intervention improved intestinal mechanical barrier integrity (**Figure S10a-b**) and increased the expression of the tight junction protein Claudin-1 and Occludin (**Figure S10c-e**). The FITC-dextran gavage assay and flow cytometry analysis of OmpA in EVs isolated from peripheral blood of wild-type mice post-I/R injury showed consistent results (**Figure S10f, Figure S11**): GLP-2 intervention reduced intestinal permeability, preserved gut barrier integrity, and effectively inhibited bacterial EV translocation.

We subsequently investigated the effects of GLP-2-mediated inhibition of bacterial EV translocation on inflammation and cardiac function. We used flow cytometry to analyze the quantity and subtypes of myeloid cells of ABX mice that received oral *E. coli* EVs, either with or without GLP-2 treatment. Our analysis revealed GLP-2 treatment significantly reduced the proportion of Ly6C^high^ monocytes/macrophages and neutrophils in both the peripheral circulation and cardiac tissue at 3 days after I/R injury. (Figure 6d-g**, Figure S12a-d**). Furthermore, flow cytometry analysis of the spleen—a reservoir for monocytes/macrophages—demonstrated that systemic monocyte/macrophage mobilization was also notably attenuated following GLP-2 treatment and the inhibition of EV translocation (**Figure S12e-f**). Additional evaluations, including immunofluorescence staining of monocytes/macrophages (CD68) in cardiac tissue (Figure 6h-i), the measurement of peripheral pro-inflammatory cytokines (Figure 6j), and the analysis of inflammatory cell infiltration (**Figure S13**), further confirmed the role of GLP-2 in mitigating systemic inflammatory responses and reducing local monocyte/macrophage infiltration triggered by gut-derived PAMPs.

We next investigated whether GLP-2 treatment could alleviate cardiac dysfunction induced by *E. coli* EVs following I/R injury. During the acute phase of I/R injury, GLP-2 treatment significantly reduced infarct size and preserved ischemic cardiac tissue by modulating the inflammatory response (Figure 6l-m). Echocardiographic assessments revealed improved ejection fraction values during the chronic repair phase with GLP-2 intervention (Figure 6k). Four weeks post-I/R surgery, GLP-2 treatment notably alleviated cardiac fibrosis (Figure 6n-o), indicating that GLP-2 not only enhances cardiac function in the acute phase but also supports long-term cardiac tissue repair. Collectively, these findings highlight that GLP-2 mitigates pro-inflammatory responses during I/R injury by inhibiting the translocation of *E. coli* EVs, thereby promoting sustained improvements in cardiac function.

## Discussion

In this study, with *E. coli* as the primary research focus, we have, for the first time, demonstrated that bacterial EVs produced after myocardial I/R injury can cross the intestinal mucosal barrier, entering the peripheral circulation and cardiac tissue. Moreover, *E. coli* EVs showed a significant correlation with circulating LPS levels. Consequently, the migration of EVs carrying PAMPs exacerbates innate immune responses—primarily mediated by monocytes/macrophages and neutrophils—thereby aggravating myocardial injury and impairing cardiac functional recovery. Notably, GLP-2 therapy targeting the intestinal mucosal barrier effectively inhibits EVs translocation, modulates acute-phase inflammatory responses, and promotes tissue repair. These findings provide novel insights and strategies for immune modulation following myocardial I/R injury.

In recent years, the concept of the "gut-heart axis" has gained increasing attention in studies exploring the relationship between cardiovascular diseases and the gut microbiome. Alterations in gut microbiota have been identified in various cardiovascular conditions, including myocardial infarction, atrial fibrillation, and hypertension.^28-30^ Notably, several studies on myocardial infarction patients have demonstrated a strong association between gut microbiota changes, cardiac function, and systemic inflammatory responses.^12^^,^^31^ Research on the "gut-heart axis" can be broadly divided into two primary directions. The first focuses on the impact of gut microbiota-derived metabolites, such as short-chain fatty acids^32^^,^^33^ and trimethylamine-*N*-oxide,^34^^,^^35^ which have been shown to play significant roles in cardiac injury and repair. Furthermore, studies have shown that in the left anterior descending artery ligation mouse model of acute myocardial infarction established in germ-free mice, cardiac function is decreased compared with that in their conventionally raised SPF controls.^36^ The second direction centers on the effects of bacterial translocation and harmful components, such as metabolic toxins and PAMPs, crossing a compromised intestinal mucosal barrier and influencing cardiac repair.^37^^,^^38^ Damage to the intestinal mucosal barrier is associated with various factors, including dysbiosis, intestinal ischemia, edema, and reduced gut motility, all of which can increase intestinal permeability.^12^^,^^20^^,^^39^ Under these conditions, exogenous PAMPs, such as LPS derived from Gram-negative bacteria, can be detected in the circulation. The prevailing hypothesis attributes this phenomenon to bacterial invasion or translocation, as some studies have observed gut-derived bacteria migrating into the bloodstream and other organs.^20^

However, bacterial products are not limited to various gut-derived metabolites. Similar to eukaryotic cells secreting EVs, bacteria also produce EVs.^16^ Gut bacteria-derived EVs can directly interact with intestinal epithelial cells, influencing processes such as autophagy and immune modulation. For example, EVs from *B. fragilis* can regulate Treg cells and anti-inflammatory cytokine production, contributing to the prevention of inflammatory bowel disease.^40^ Additionally, EVs from *P. pentosaceus* and *Lactobacillus* species have been shown to inhibit cellular and humoral immune responses as well as macrophage polarization, thereby modulating host immunity.^41^^,^^42^ Due to their nanoscale size, bacterial EVs can more easily cross the intestinal mucosal barrier compared to whole bacteria. Furthermore, EVs possess the capacity to exert effects at distant sites within the body. Studies have demonstrated that bacterial EVs play a critical role in gut-brain communication, as they can traverse the blood-brain barrier and accumulate in brain tissues, contributing to diseases such as depression, Alzheimer's disease, and atherosclerosis.^19^^,^^43^^,^^44^ Despite the aforementioned research progress, the role of bacterial EVs in acute conditions like myocardial infarction remains unexplored, which leaves a significant gap in our understanding of their potential functions in such diseases. This study aims to investigate the translocation of bacterial EVs and determine whether they serve as a vehicle for LPS entry into circulation. Based on our prior research,^20^ *Escherichia* is recognized as an important member of the intestinal microbiota, with a high abundance. Moreover, the abundance of *Escherichia* in patients with acute myocardial infarction also shows a significant increase.^45^ Therefore, it is an ideal model for this study. Meanwhile, *Lactobacillus*, recognized as a probiotic, was selected as a negative control. Intestinal Lactobacillus colonies have been reported to exert protective effects on the heart by modulating atheroinflammatory responses, cholesterol metabolism, and oxidative stress. Specifically, *L. plantarum* has been shown to have no harmful effects on mice in prior studies,^42^^,^^43^ further supporting its suitability as a control in this study. Subsequent proteomic analyses in this study confirmed the presence of LPS and other PAMPs in *E. coli* EVs. Although we cannot determine the proportion of LPS transferred via EVs relative to the total LPS pool, a strong correlation was observed between *E. coli* EVs and circulating LPS levels, suggesting their critical involvement in LPS translocation following myocardial infarction. Moreover, due to EVs’ inherent immune escape capacity,^46^ LPS derived from EVs may exert a more profound impact on the host—findings we will further elucidate in subsequent studies.

Given that bacterial EVs carry various PAMPs, including LPS, they inevitably modulate multiple immune cell populations. As a major pathogenic factor of Gram-negative bacteria, LPS promotes inflammation through interaction with the pattern recognition receptor TLR4, affecting neutrophils, monocytes/macrophages, and T cells.^13^^,^^20^^,^^47^ Our in vitro experiments also confirmed that *E. coli* EVs can activate the TLR4 signaling pathway. Extensive studies have demonstrated that subsequent LPS-TLR4 signal transduction mediates intracellular signaling cascades through both MyD88-dependent and MyD88-independent pathways.^48^ Monocytes/macrophages, as key innate immune cells, rapidly infiltrate ischemic myocardium during the early stages of myocardial infarction, transitioning to a pro-inflammatory phenotype.^49^^,^^50^ Gut-derived EVs exacerbate and accelerate this inflammatory process, leading to excessive secretion of inflammatory cytokines. Our study confirmed that *E. coli* EVs enhance the mobilization of monocytes/macrophages from the spleen, their infiltration into cardiac tissue, and their transition to a pro-inflammatory phenotype, driving the production of various pro-inflammatory cytokines. Effective repair of the heart following myocardial infarction and reperfusion requires not only the timely initiation of inflammation to clear necrotic cells and tissues but also the timely resolution of the inflammatory response. Numerous studies, including those from our team, have demonstrated that the pro-inflammatory phenotype of monocytes/macrophages following myocardial I/R injury is closely associated with left ventricular function and remodeling.^7^^,^^20^ Proper regulation of the transition between pro-inflammatory and anti-inflammatory phenotypes is critical for tissue repair after cardiac injury. Excessive inflammation during the acute phase of myocardial injury inevitably exacerbates cardiac damage. In the *E. coli* EV group, we observed larger infarct areas, excessive myocardial fibrosis, and worse cardiac function, consistent with the detrimental effects of unchecked inflammatory responses. Of course, besides LPS, EVs also carry many other exogenous molecules. Delivering virulence factors to host cells is the most important function of bacterial EVs, and they transport more than just LPS. For example, vacuolating cytotoxin A produced by *Helicobacter pylori* can be continuously released via EVs to induce mild gastritis, creating conditions for the persistent survival of *Helicobacter pylori* in the host.^51^ EVs derived from *P. aeruginosa* contain a complex entity composed of flagellin, LPS, and other proteins, which can induce inflammatory responses in host cells.^52^ Novel virulence factors, including PagK and PagJ, have been identified in EVs from *Salmonella enterica*. Therefore, the specific mechanisms and downstream signaling pathways need to be further elucidated in subsequent studies.^53^

Cardiovascular diseases can induce intestinal mucosal barrier dysfunction, which correlates with LPS invasion.^12^^,^^54^^,^^55^ In this study, we employed GLP-2 to enhance intestinal barrier integrity and function.^20^^,^^27^ Our findings indicate that GLP-2 treatment can partially mitigate EV translocation, thereby attenuating inflammatory responses and promoting myocardial repair. Previous studies have also ruled out the direct effects of GLP-2 on inflammatory cells.^20^ The underlying molecular mechanisms warrant further in-depth investigation in subsequent studies.

In summary, our study is the first to demonstrate the impact of gut bacterial EVs on the pathological process of myocardial I/R injury. We identified that I/R injury promotes the translocation of bacterial EVs across the gut barrier and their subsequent migration to distant organs. These findings provide novel insights into the interplay between the cardiovascular system and the gut microbiota. Furthermore, our results offer novel theoretical support for the therapeutic potential of GLP-2 in the management of MI patients.

## Supplementary Material

Supplemental_Material__3_.docx Supplemental Material

Table S1 Proteomic analysis Ecoli EVs.xls Table S1 Proteomic analysis Ecoli EVs.xls

Table S3 Characteristics of STEMI Patients.xlsx Table S3 Characteristics of STEMI Patients.xlsx

Table S2 Proteomic analysis of Lplantarum EVs.xlsx Table S2 Proteomic analysis of Lplantarum EVs.xlsx

## Data Availability

The proteomics results of *E. coli* and *L. plantarum* EVs have been included in the supplementary materials and uploaded to a public database (https://data.4tu.nl/private_datasets/fwCME4mSN6EklQqCu4UzbOsbbyCr39UWcZp3pPEU7-c). Other data that support the findings of this study are available from the corresponding author upon reasonable request.

## Abbreviations

I/R: ischemia/reperfusion
PAMPs: pathogen-associated molecular patterns
LPS: lipopolysaccharides
EVs: extracellular vesicles
E. *coli*: Escherichia coli
L. *plantarum*: Lactobacillus plantarum
ABX mice: mixture antibiotic-treated mice
FACS-beads: flow cytometry-based protocol to label proteins on beads-bound EVs
STEMI: ST-segment elevation myocardial infarction
WW/DW: wet-to-dry weight ratio
HW/TL: heart weight-to-tibia length ratio
cTnT: cardiac troponin T
Myo: myoglobin
GLP-2: glucagon-like peptide 2
