# Supplementary material for: Invasion of gut-derived escherichia coli extracellular vesicles exacerbates myocardial ischemia/reperfusion injury
Source: Gut Microbes. 2026 Feb 28;18(1):2635818. doi: 10.1080/19490976.2026.2635818 (PMC12959223; doi:10.1080/19490976.2026.2635818)
Supplement: Supplemental_Material__3_.docx — Supplemental Material [file KGMI_A_2635818_SM1900.docx]

**Invasion of Gut-Derived Escherichia coli Extracellular Vesicles Exacerbates Myocardial Ischemia/Reperfusion Injury**

**Running title**: Gut Bacterial EVs Aggravate Myocardial I/R Injury

Junzhuo Wang ^1^*, Ke Hu ^2^*, He Lu ^3 4^, Ke Chen ^5^, Jian Zhang ^6^，Shaojun Wu ^1^, Lina Kang ^1·#^, Jun Xie ^7#^, Biao Xu ^1·#^

^1^ Jiangsu Key Laboratory for Cardiovascular Information and Health Engineering Medicine, Department of Cardiology, Nanjing Drum Tower Hospital, Affiliated Hospital of Nanjing University Medical School, Nanjing, People's Republic of China;

^2^The Affiliated Drum Tower Hospital of Nanjing Medical University, Nanjing, People's Republic of China;

^3^Nanjing Drum Tower Hospital, Drum Tower Clinical College, Nanjing University of Chinese Medicine, No. 321 Zhongshan Road, Nanjing, People's Republic of China;
^4^Department of Cardiology, The People's Hospital of Jiawang District of Xuzhou, Xuzhou, People's Republic of China a;

^5^Department of Vascular Surgery, Drum Tower Hospital, Affiliated Hospital of Nanjing University Medical School, Nanjing, People's Republic of China;

^6^Department of Cardiology, National Cardiovascular Disease Regional Center for Anhui, the First Affiliated Hospital of Anhui Medical University, Hefei, People's Republic of China;

^7^ Department of Science and Technology, Nanjing Drum Tower Hospital, Affiliated Hospital of Nanjing University Medical School, Nanjing, People's Republic of China.

^#^Corresponding author: Biao Xu, MD, PhD, Department of Cardiology, The Affiliated Hospital of Nanjing University Medical School, No. 321 Zhongshan Road, Nanjing, Jiangsu, China. E-mail: xubiao62@nju.edu.cn. Jun Xie PhD, Department of Cardiology, The Affiliated Hospital of Nanjing University Medical School, No. 321 Zhongshan Road, Nanjing, Jiangsu, China. Email: xiejun@ahmu.edu.cn. And Lina Kang, PhD, Department of Cardiology, The Affiliated Hospital of Nanjing University Medical School, No. 321 Zhongshan Road, Nanjing, Jiangsu, China. E-mail: kanglina@njglyy.com

*These authors contributed equally to this work

**Supplemental Material**


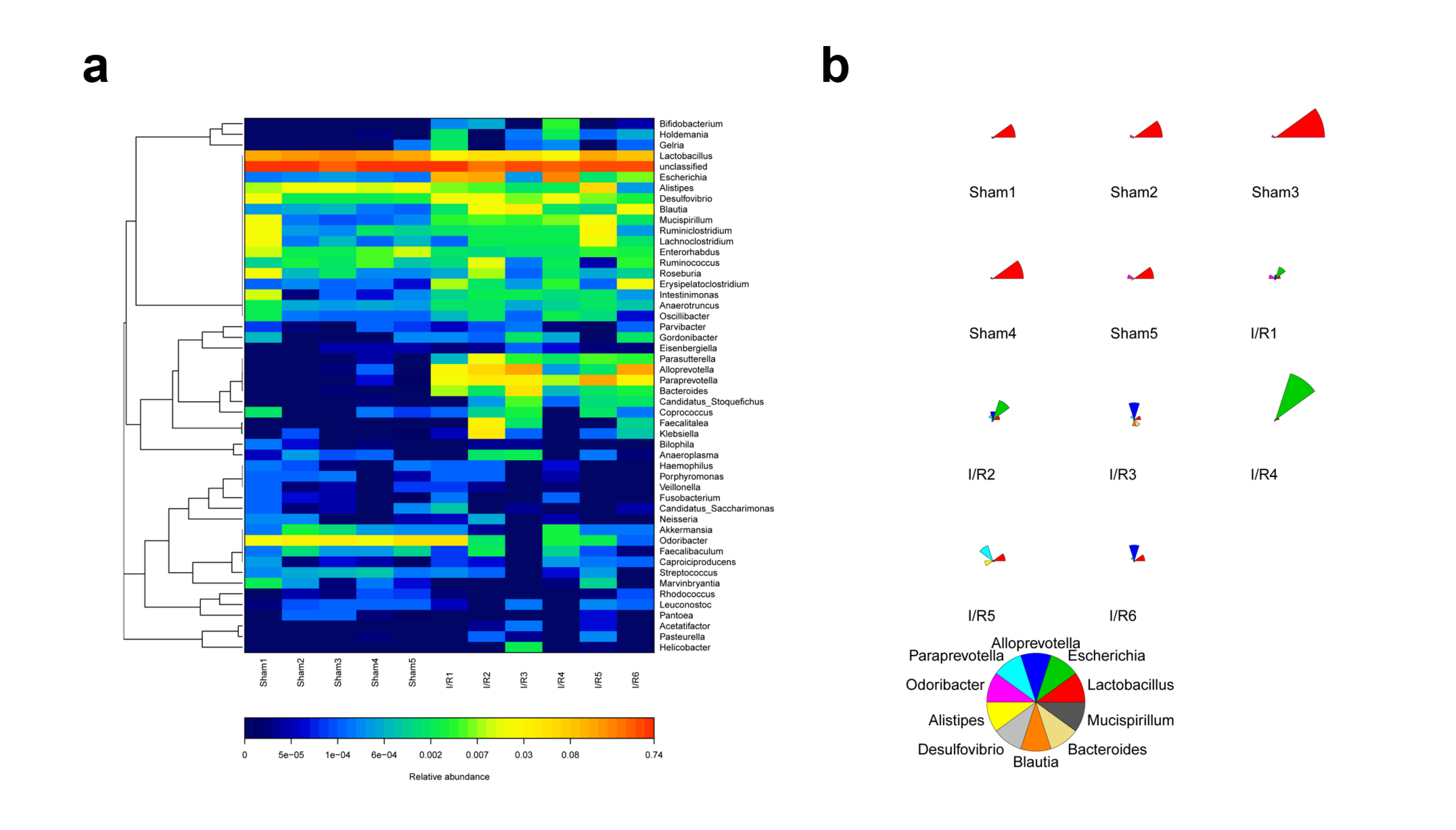
**Supplementary Figure 1**

**Figure S1. a.** Genus-level abundance heatmap between the sham and I/R groups. **b.** Relative abundance distribution comparison of the top 10 most abundant genera between the sham and I/R groups

**Supplementary Figure 2**

**
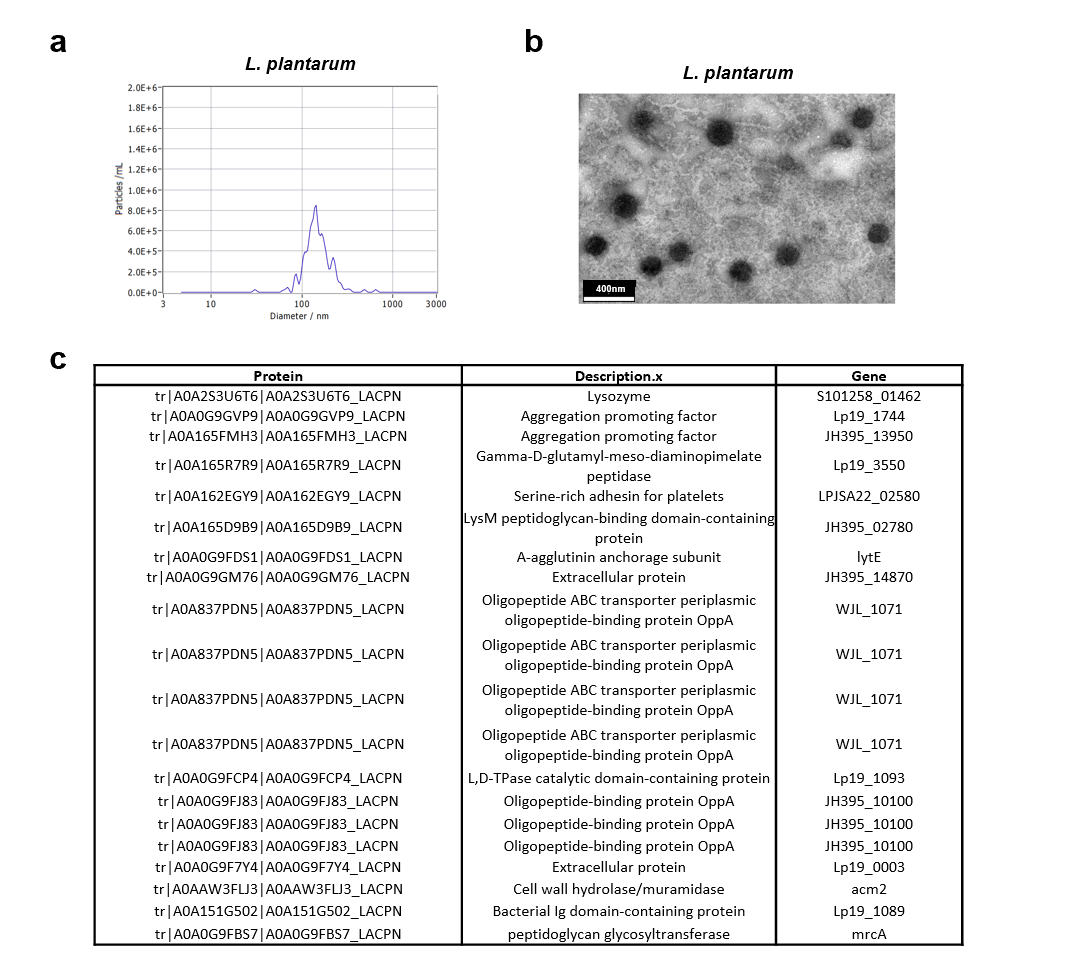
**

**Figure S2.** Characterization of *L. plantarum* EVs. Transmission electron microscopy images (**a**) and Nanoparticle tracking analysis (**b**) of *E. coli* EVs. Scale bar: 400nm. **c**. Top 20 most abundant proteins in *E. coli* EVs.

**Supplementary Figure 3**


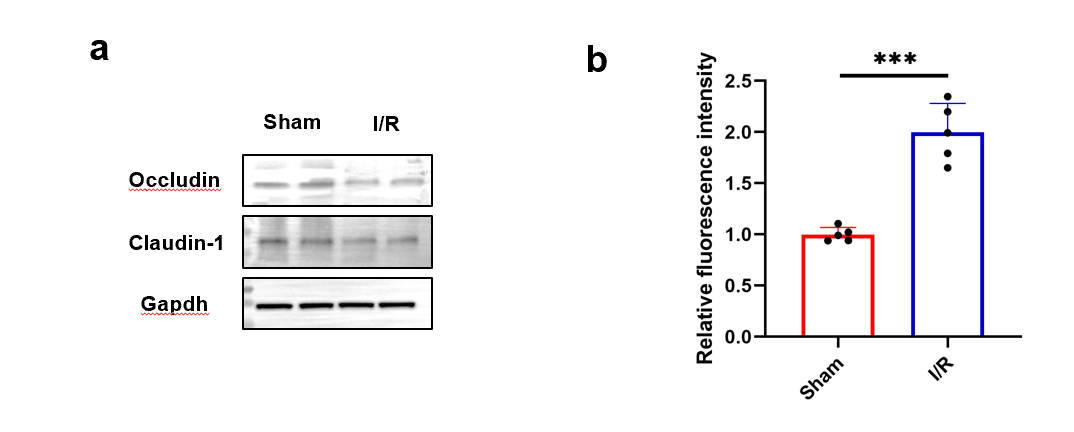


**Figure S3. a.** Western blot detection of Occludin and Claudin-1 proteins in intestinal mucosa. **b.** FITC-dextran permeability assay in mice with cardiac I/R injury and sham-operated mice (n = 5 mice). (ns, not significant, *P < 0.05, **P < 0.01, ***P < 0.001)

**Supplementary Figure 4**


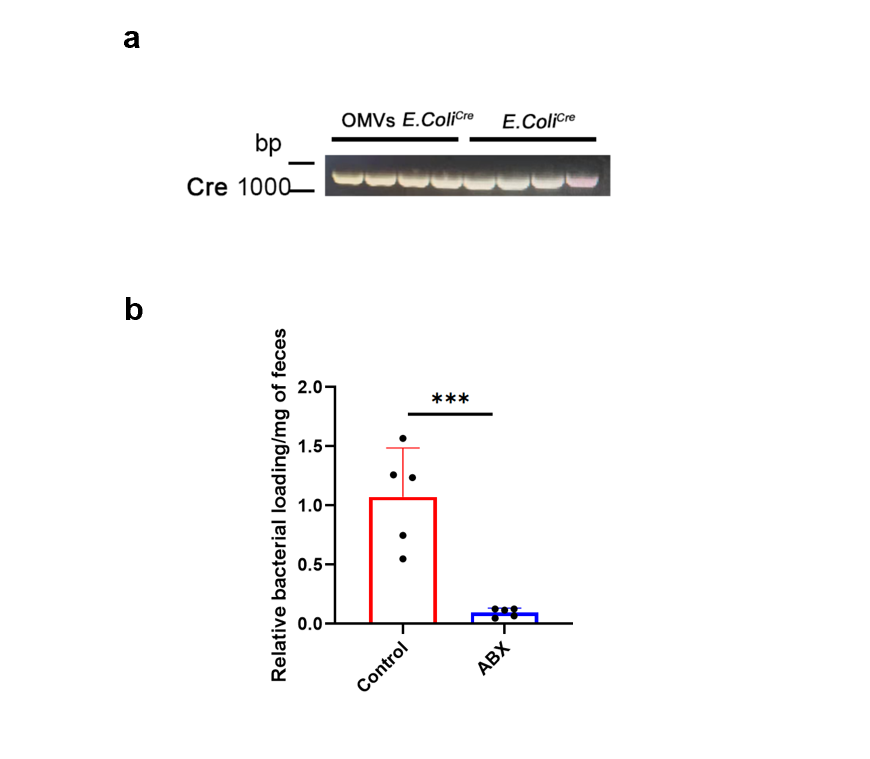


**Figure S4. a.** RT-PCR analysis of Cre DNA from the whole bacterial lysate and purified *E. coli*^Cre^ isolated by ultracentrifugation. **b.** Quantification of bacterial load in fecal samples from both ABX-treated and control mice was performed (n = 5 mice). (ns, not significant, *P < 0.05, **P < 0.01, ***P < 0.001)

**Supplementary Figure 5**


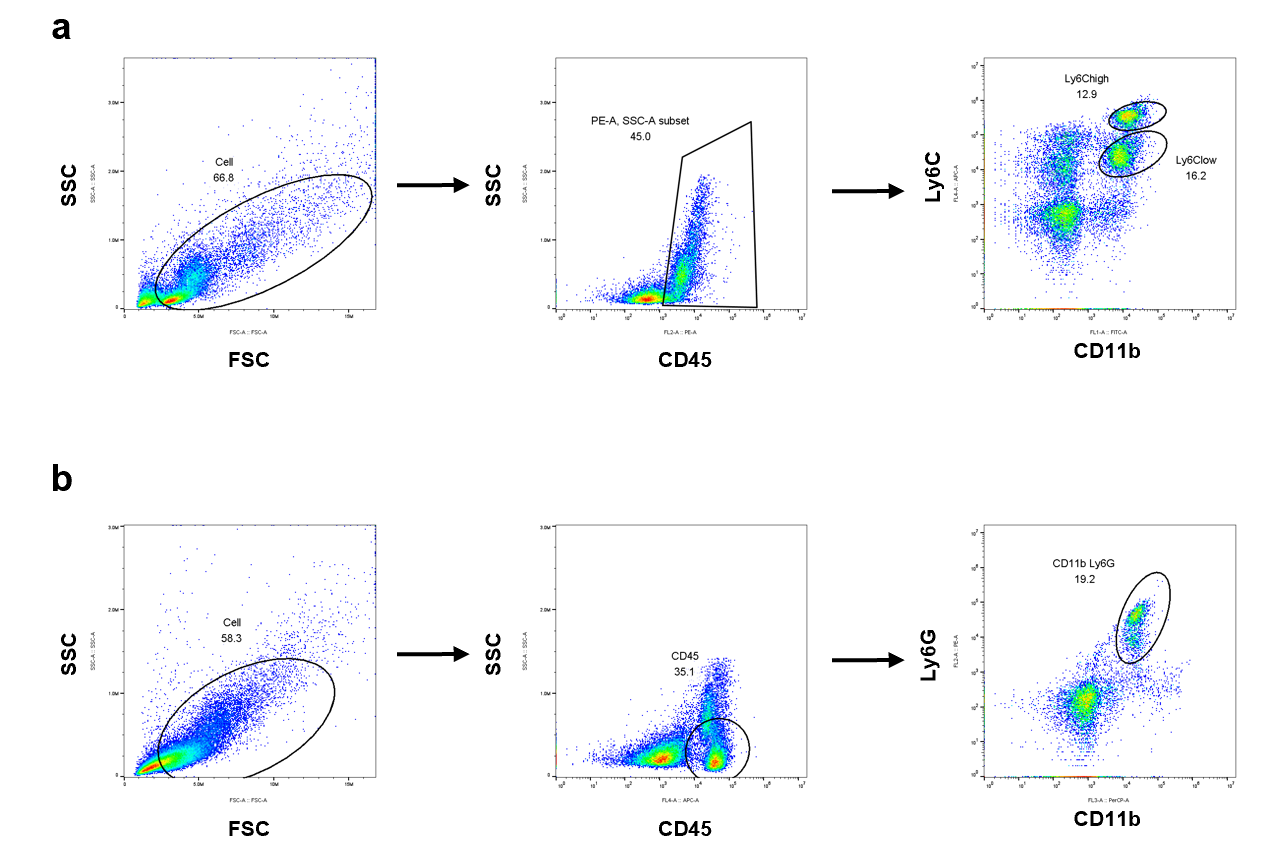


**Figure S5. a.** Gating strategy for Ly6C^high^ monocytes and Ly6C^low^ monocytes/macrophages (CD45^+^CD11b^+^). **b**. Gating strategy for neutrophils (CD45^+^CD11b^+^Ly6G^+^)

**Supplementary Figure 6**


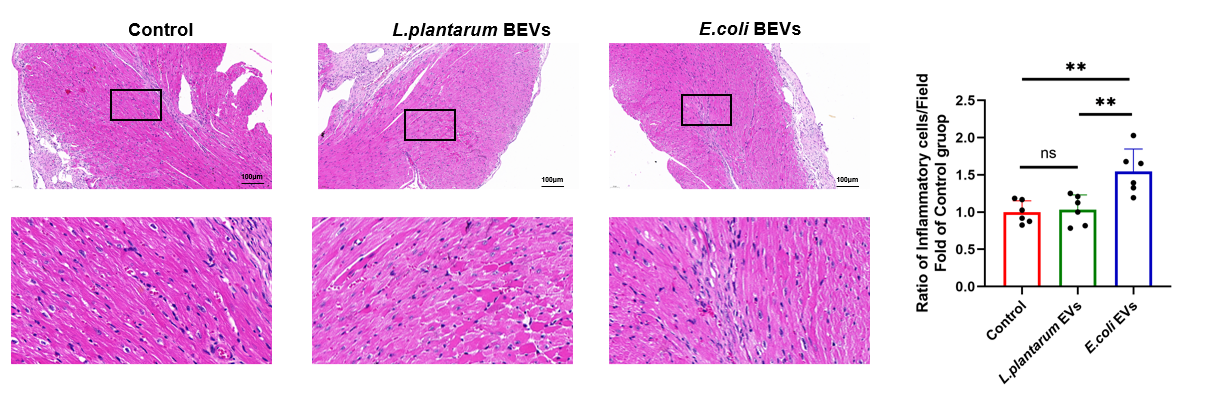


**Figure S6.** Representative HE staining images and magnified views of ischemic hearts from mice treated with *E. coli* EVs, *L. plantarum* EVs, or control groups 3 days post-surgery, showing inflammation and immune cell infiltration (n=6 mice). Scale bar: 100µm. (ns, not significant, *P < 0.05, **P < 0.01, ***P < 0.001)

**Supplementary Figure 7**


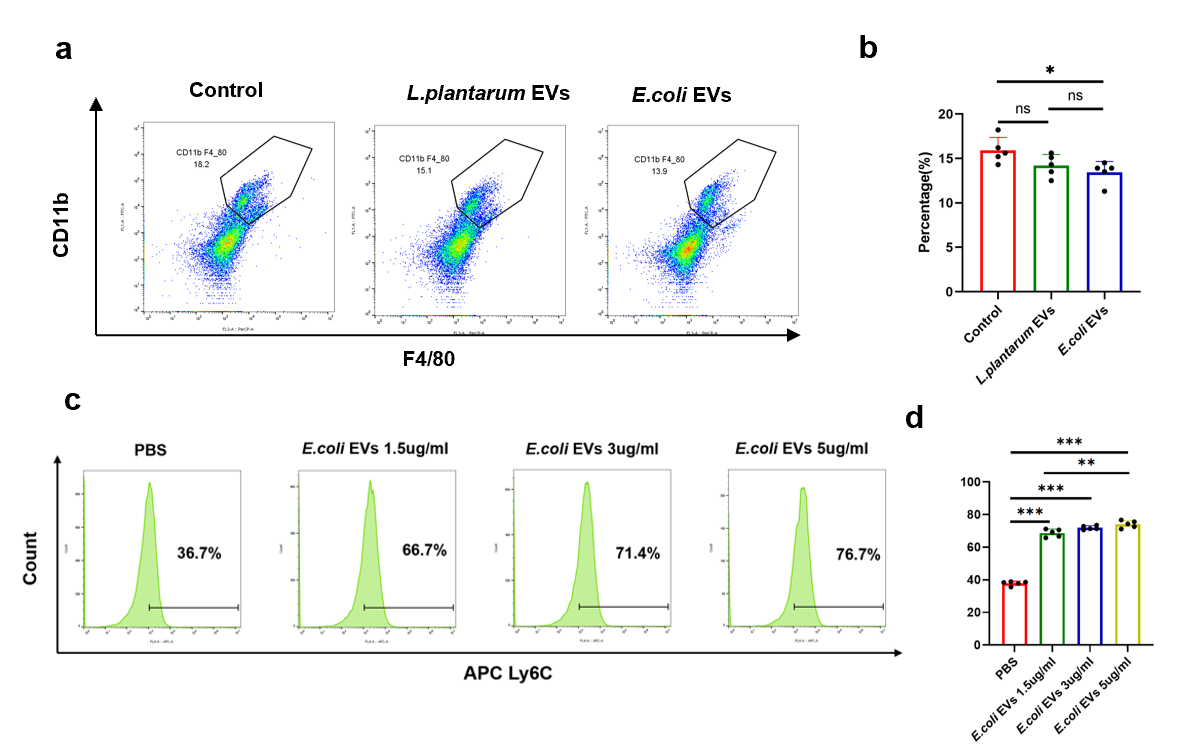


**Figure S7. a-b.** Representative flow cytometry plots showing monocytes/macrophages (F4/80^+^CD11b^+^) in spleen at 3 days after oral gavage and myocardial I/R injury, along with quantitative analysis (n = 5 mice). **c-d.** Representative histograms and statistical results of Ly6C expression in RAW264.7 cells treated with 1.5µg/ml, 3 µg/ml and 5 µg/ml *E. coli* EVs for 24 hours, as detected by flow cytometry (n = 5). (ns, not significant, *P < 0.05, **P < 0.01, ***P < 0.001)

**Supplementary Figure 8**


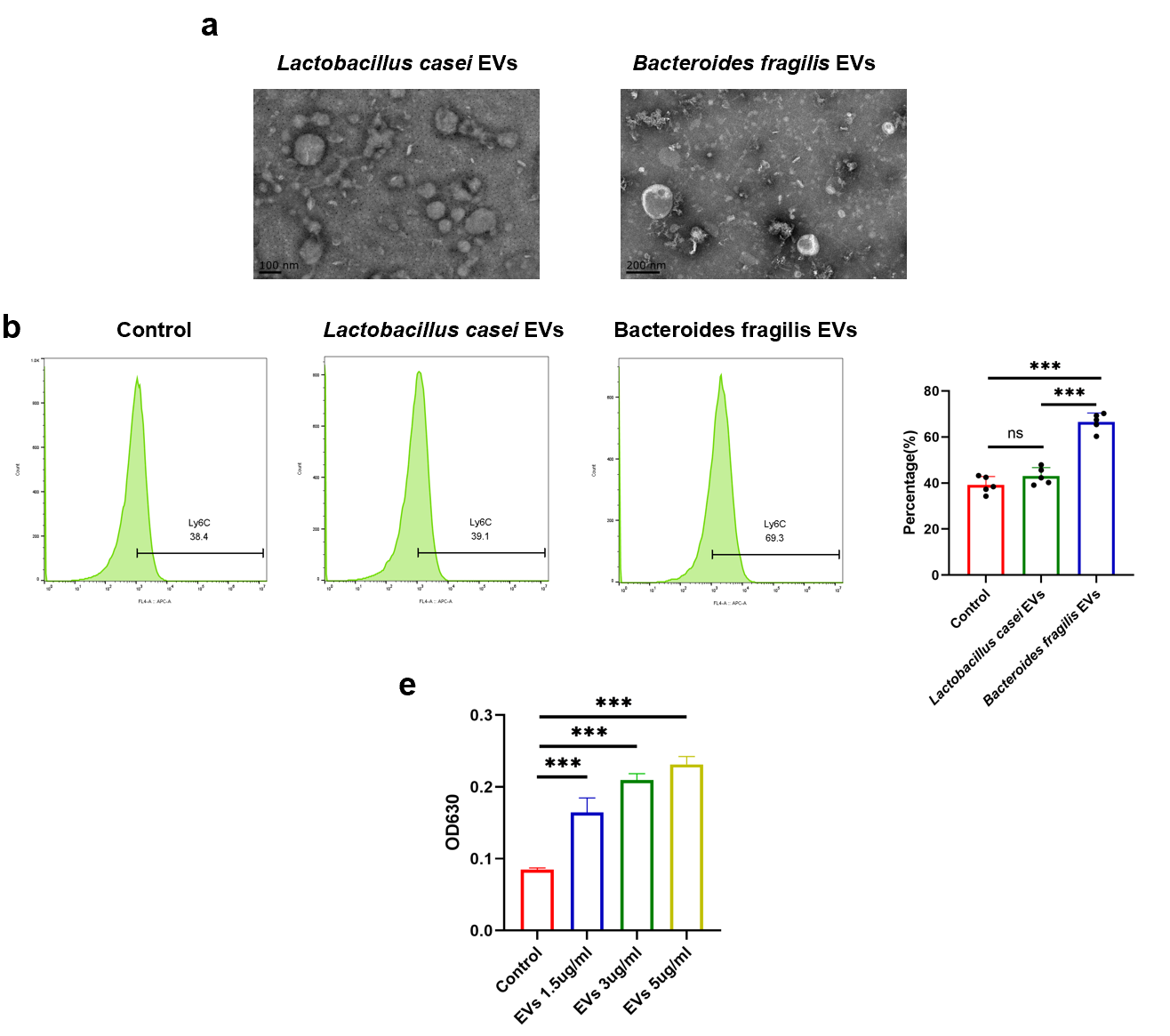


**Figure S8 a.** Transmission electron microscopy images of *E. coli* EVs. Scale bar: 200nm. **b**. Representative histograms and statistical results of Ly6C expression in RAW264.7 cells treated with *Lactobacillus casei* EVs and *Bacteroides fragilis* EVs for 24 hours, as detected by flow cytometry (n = 5). **c**. Detection of TLR4 Signaling Pathway Activity in HEK-Blue™ mTLR4 Cells Treated with Bacterial EVs (n = 5). (ns, not significant, *P < 0.05, **P < 0.01, ***P < 0.001)

**Supplementary Figure 9**


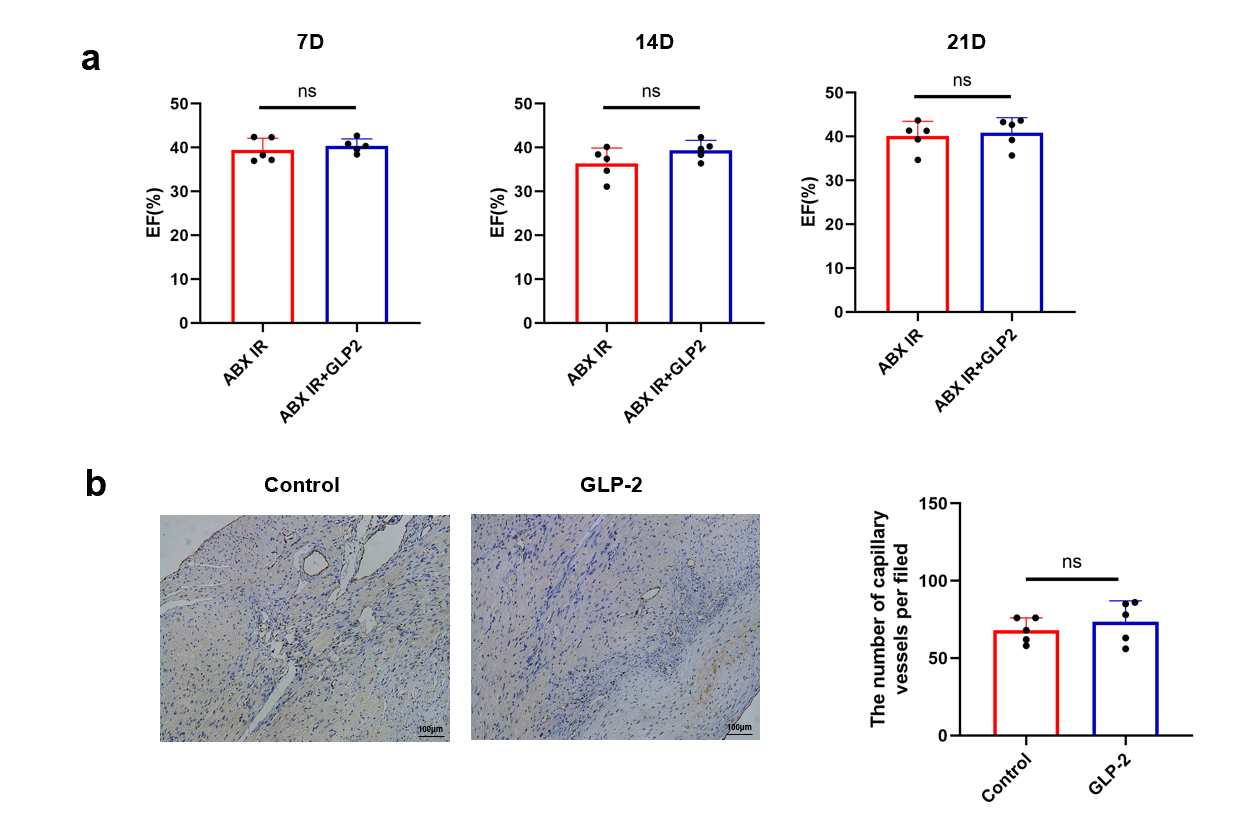


**Figure S9 a.** Statistical results of left ventricular ejection fraction (EF) at different time points after GLP-2 treatment in ABX-treated mice (n=5 mice). **b**. Representative images of CD31 immunohistochemistry in the myocardial infarct area and statistical analysis results of neovascularization in ABX-treated mice after GLP-2 treatment (n=5 mice). (ns, not significant, *P < 0.05, **P < 0.01, ***P < 0.001)

**Supplementary Figure 10**


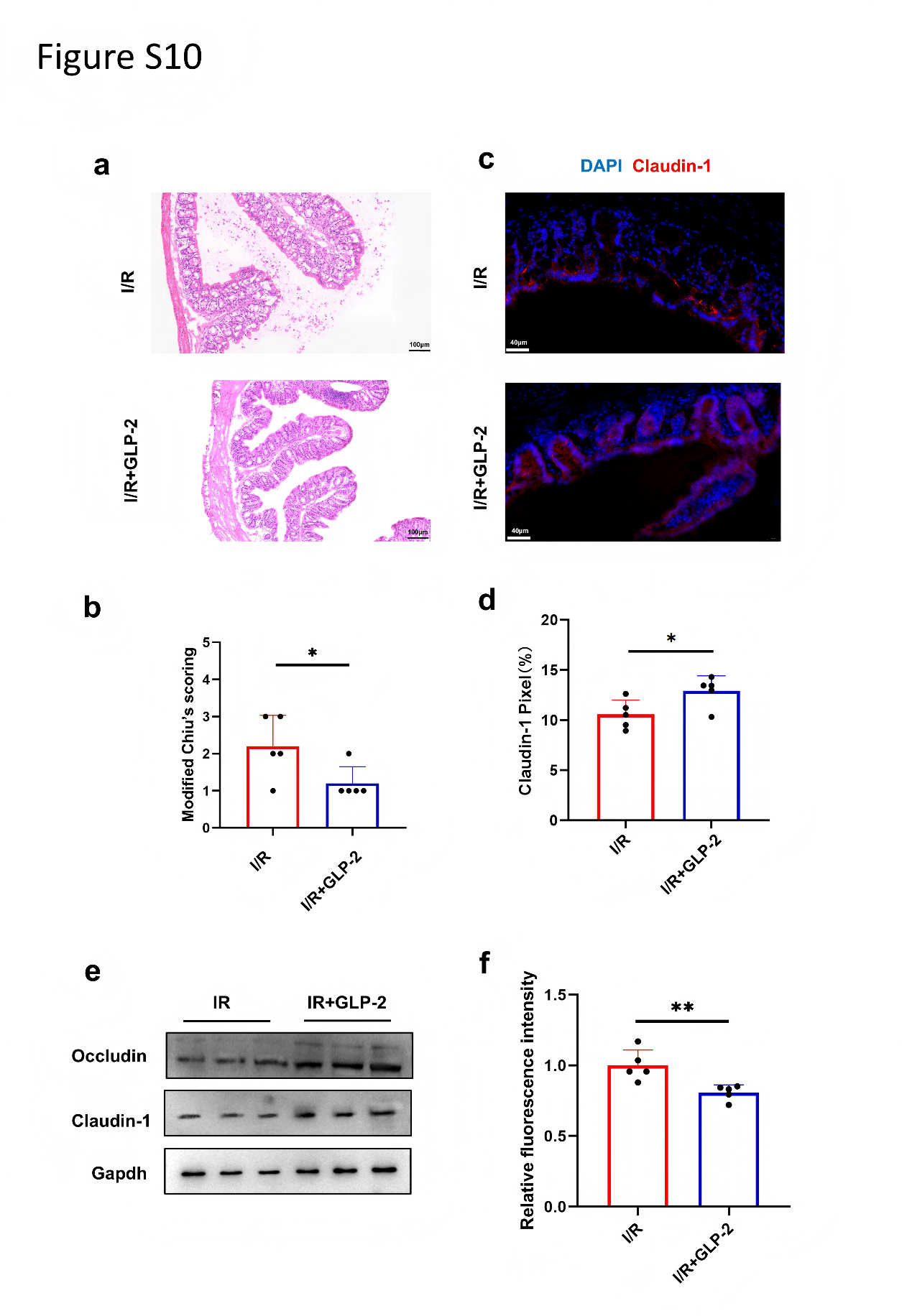


**Figure S10.** **a-b.** Representative HE staining images of intestinal tissue following I/R injury, with or without GLP-2 intervention, along with modified Chiu's Score quantitative results (n=5 mice). Scale bar: 100µm. **c-d.** Representative immunofluorescence images of ventricular claudin-1 (red) and DAPI (blue) staining in Rosa26.tdTomato reporter mice colonized with *E. coli*^Cre^ following I/R injury, with or without GLP-2 intervention, along with quantitative results (n=5 mice). Scale bar: 40µm. **e**. Western blot detection of Occludin and Claudin-1 proteins in intestinal mucosa with or without GLP-2 treatment. **f.** FITC-dextran permeability assay in mice with cardiac ischemia-reperfusion (I/R) injury: with or without GLP-2 treatment. (ns, not significant, *P < 0.05, **P < 0.01, ***P < 0.001)

**Supplementary Figure 11**


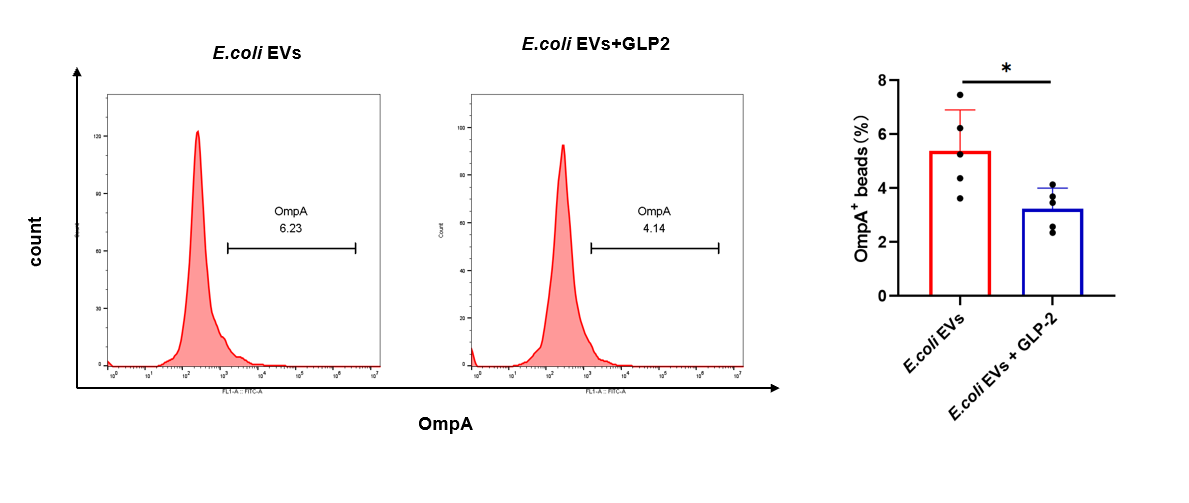


**Figure S11.** Representative histograms showing the levels of OmpA in EVs extracted from mouse peripheral blood, with or without GLP-2 intervention, along with statistical results (n=5 mice). (ns, not significant, *P < 0.05, **P < 0.01, ***P < 0.001)

**Supplementary Figure 12**


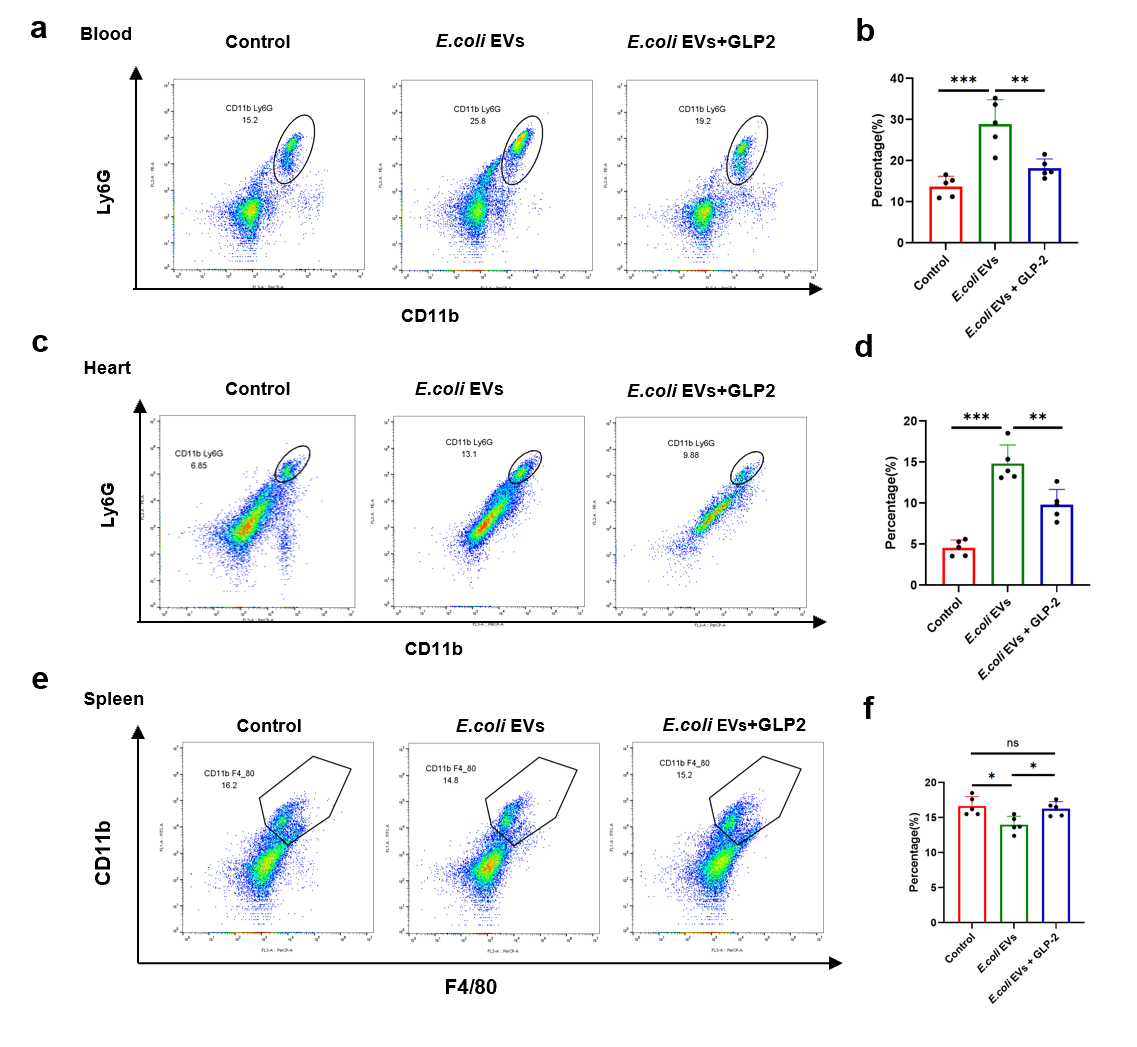


**Figure S12. a-b.** Representative flow cytometry plots showing neutrophils (CD45+CD11b+Ly6G+) in peripheral blood, with or without GLP-2 intervention, along with quantitative analysis (n = 5 mice). **c-d.** Representative flow cytometry plots showing neutrophils (CD45+CD11b+Ly6G+) in cardiac tissue, with or without GLP-2 intervention, along with quantitative analysis (n = 5 mice). **e-f.** Representative flow cytometry plots showing monocytes/macrophages (F4/80^+^CD11b^+^) in spleen, with or without GLP-2 intervention, along with quantitative analysis (n = 5 mice). (ns, not significant, *P < 0.05, **P < 0.01, ***P < 0.001)

**Supplementary Figure 13**


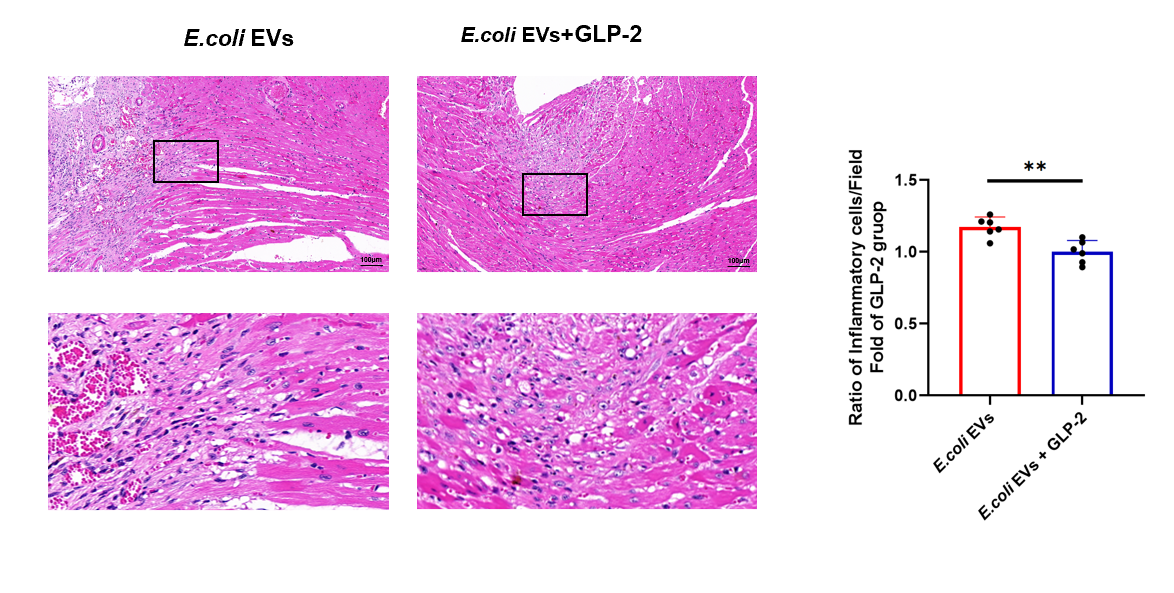


**Figure S13.** Representative HE staining images and magnified views of ischemic hearts from mice treated with or without GLP-2 3 days post-surgery, showing inflammation and immune cell infiltration (n=6 mice). Scale bar: 100µm. (ns, not significant, *P < 0.05, **P < 0.01, ***P < 0.001)

**Supplemental Methods**

**Study Population**

This observational case-control study enrolled 16 ST-segment elevation myocardial infarction (STEMI) patients admitted to the First Affiliated Hospital of Anhui Medical University and 10 age- and gender-matched healthy controls without cardiovascular or cerebrovascular diseases. All STEMI patients were diagnosed according to current European guidelines and successfully underwent primary percutaneous coronary intervention. Patient exclusion criteria comprised: elderly patients (>80 years old); critically ill status due to STEMI; sustained ventricular tachycardia and ventricular fibrillation post-STEMI; definitive mechanical complications (ventricular septal rupture, papillary muscle rupture, or cardiac rupture); severe cardiogenic shock unresponsive to vasopressors; renal failure (glomerular filtration rate <30 ml/min/1.73m²); acute infectious diseases within 2 weeks prior to enrollment or chronic inflammatory diseases; immunocompromised status; neuropsychiatric disorders; malignancy with expected survival <1 year; hepatic failure; known hypersensitivity to study drug components; pregnancy, lactation, or planned pregnancy during study period; and any other investigator-determined contraindications to clinical trial participation. Demographic, clinical, and biological characteristics of STEMI patients were recorded. Baseline complete blood count and biochemical analyses were performed upon hospital admission. Blood samples for LPS and bacterial EVs detection were collected 48 hours after admission and stored at -80°C until analysis.

**Bacteria culture**

The recombinant *E. coli^Cre^* strain (DH5α/pGEX-4T-1) containing both Cre recombinase and ampicillin resistance genes was constructed using the pGEX-4T-1 vector, which was kindly provided by Vigene Biosciences. *E. coli^GFP^* (ATCC 25922GFP), *Lactobacillus plantarum* (BNCC336469, BeNa), Bacteroides fragilis (BNCC371828, BeNa) and *Lactobacillus casei* (BNCC134415, BeNa) were obtained as commercially available strains and utilized directly in the experiments.

*E. coli^Cre^* and *E. coli^GFP^* were cultured in LB broth supplemented with ampicillin (100 mg/L) as a selective antibiotic. For solid medium preparation, 15 g/L agar powder was added to the broth. Bacterial cultures were grown in baffled flasks at 37°C with continuous shaking (150 rpm) in the presence of isopropyl β-D-1-thiogalactopyranoside (IPTG) for induction. Overnight cultures were used to inoculate fresh medium to an initial optical density (OD600) of approximately 0.1, followed by incubation at 37°C until the cultures reached exponential growth phase (OD600 ~ 1.0). Bacterial cells were subsequently harvested by centrifugation (4,000 × g, 10 min, 4°C). *Lactobacillus plantarum*, *Bacteroides fragilis* and *Lactobacillus casei* were cultured anaerobically in de Man, Rogosa and Sharpe (MRS) medium using an anaerobic chamber system.

**EVs isolation and identification**

EVs were isolated using differential ultracentrifugation. Briefly, bacterial culture supernatants or serum were sequentially centrifuged at 3,000 g for 25 minutes and 10,000 g for 1 hour at 4°C to remove cellular debris and dead cells. The clarified supernatant was then subjected to ultracentrifugation at 100,000 g for 3 hours at 4°C. The pelleted EVs were resuspended in phosphate-buffered saline (PBS) for subsequent analysis.

EV characterization was performed using multiple complementary approaches. Morphological examination was conducted by transmission electron microscopy (JEM-1011, Japan). Nanoparticle tracking analysis was performed using ZETA-VIEW (Particle Metrix) to determine particle size distribution. EV identity was confirmed through detection of specific marker proteins OmpA (orb422682, Biorbyt) and OmpC (orb686344, Biorbyt) by western blot analysis.

Protein quantification was performed using the bicinchoninic acid (BCA) assay (Thermo Scientific, 23225). A strong correlation between protein concentration determined by BCA assay and particle count measured by nanoparticle tracking analysis (NTA) validated the use of protein concentration as a reliable indicator of EV quantity in subsequent experiments [1].

**Liquid chromatography‐mass spectrometry (LC‐MS) analysis**

The LC-MS analysis was performed by Novogene Co., Ltd. (Beijing, China) using their standardized protocols. Briefly, the technical procedure included the following steps: Samples were retrieved from -80°C storage and transferred to 1.5 mL microcentrifuge tubes. An appropriate volume of DB protein lysis buffer (8 M urea, 100 mM TEAB, pH 8.5) was added, followed by vortex mixing and ice-water bath sonication for 5 min to ensure complete lysis. The lysate was centrifuged at 12,000 g for 15 min at 4°C. The supernatant was collected and treated with 10 mM DTT at 56°C for 1 h, followed by alkylation with iodoacetamide (IAM) in the dark at room temperature for 1 h.

Protein concentration was determined using the Bradford Protein Assay Kit (Beyotime Biotechnology) according to the manufacturer's protocol. A BSA standard curve was prepared with concentrations ranging from 0 to 0.5 µg/µL. Samples and standards were loaded in triplicate onto a 96-well plate, adjusted to 20 µL with distilled water, and mixed with 180 µL G250 dye. After 5 min incubation at room temperature, absorbance was measured at 595 nm. Protein concentration was calculated based on the standard curve. Protein samples (20 µg) were separated by 12% SDS-PAGE under reducing conditions (80 V for 20 min in stacking gel and 120 V for 90 min in separating gel). Gels were stained with Coomassie Brilliant Blue R-250 and destained until clear bands were visible.

For in-solution digestion, protein samples were adjusted to 100 µL with DB lysis buffer and digested with trypsin in 100 mM TEAB buffer at 37°C for 4 h. Additional trypsin and CaCl2 were added for overnight digestion. The pH was adjusted to <3 with formic acid, and samples were centrifuged at 12,000 g for 5 min at room temperature. The supernatant was desalted using C18 columns, washed three times with washing buffer (0.1% formic acid, 3% acetonitrile), and eluted with elution buffer (0.1% formic acid, 70% acetonitrile). The eluate was collected and lyophilized.

Mobile phases were prepared as follows: Phase A (100% water, 0.1% formic acid) and Phase B (80% acetonitrile, 0.1% formic acid). Lyophilized samples were reconstituted in 10 µL Phase A, centrifuged at 14,000 g for 20 min at 4°C, and 1 µg of supernatant was injected for LC-MS/MS analysis.Chromatographic separation was performed using an EASY-nLC™ 1200 UHPLC system equipped with a homemade pre-column (4.5 cm × 75 µm, 3 µm) and analytical column (15 cm × 150 µm, 1.9 µm). Mass spectrometry was conducted using a Q Exactive™ HF-X mass spectrometer with a Nanospray Flex™ (ESI) ion source. Instrument parameters were set as follows: ion spray voltage, 2.1 kV; ion transfer tube temperature, 320°C; full scan range, m/z 350-1500; resolution, 60,000 (at m/z 200); maximum IT, 20 ms; AGC target, 3e6. Data-dependent acquisition was performed with the following settings: top 40 most intense ions selected for HCD fragmentation; MS2 resolution, 15,000 (at m/z 200); maximum IT, 45 ms; AGC target, 1e5; normalized collision energy, 27%; intensity threshold, 2.2e4; dynamic exclusion, 20 s.

Raw data were processed using Proteome Discoverer 2.2 (Thermo Scientific) against the 1131342.fasta database (4634 sequences). Search parameters included: precursor mass tolerance, 10 ppm; fragment mass tolerance, 0.02 Da; fixed modification, carbamidomethylation of cysteine; variable modifications, methionine oxidation and N-terminal acetylation; maximum missed cleavages, 2. The false discovery rate (FDR) was set to 1% at both peptide and protein levels. Only high-confidence PSMs (≥99% confidence) and proteins with at least one unique peptide were retained for further analysis.

**Animal experimental protocol**

C57BL/6J mice were obtained from the Model Animal Research Center of Nanjing University, while C57BL/6J and R26-tdTomato (JAX: 007909) mouse lines were purchased from Shanghai Biomodel Organism Co., Ltd. Male mice were housed under specific pathogen-free (SPF) conditions with a 12-hour light/dark cycle and provided ad libitum access to autoclaved standard laboratory chow and water. Animals were randomly assigned to treatment groups and individually housed post-surgery to prevent microbial exchange through coprophagy. All surgical procedures and cage changes for germ-free mice were performed in sterilized laminar flow microbiological safety cabinets. Experimental group sizes are indicated in figure legends. Investigators performing data analysis were blinded to treatment conditions.

Antibiotic Treatment and Microbiota Depletion: To deplete gut microbiota, SPF mice received 200 µL of an antibiotic cocktail via oral gavage twice daily for 6 consecutive days. The cocktail consisted of metronidazole (1 mg/mL, Cat Number: 443-48-1, ChengYi Pharmaceutical, Zhejiang, China), vancomycin (0.5 mg/mL, Cat Number: H20080356, Eli Lilly Japan K.K.), ampicillin (1 mg/mL, Cat Number: 7177-48-2, Qilu pharmaceutical, Shandong, China), and gentamicin (1 mg/mL, Cat Number: 1403-66-3, TianFang Pharmaceutical, Shanghai, China) [2]. Animals receiving this treatment were designated as ABX mice.

Microbiota Reconstitution and Experimental Interventions: For microbiota transplantation, ABX mice were switched to selective antibiotic treatment with ampicillin (1 g/L) in drinking water on day 7. From days 8-11, mice received daily oral gavage of *E. coli^Cre^* and *E. coli^GFP^* (6×10^9^). To validate the efficacy of antibiotic treatment, we performed quantitative assessment of fecal bacterial abundance at post-antibiotic treatment (day 6). To enhance intestinal barrier permeability, a subset of mice received intraperitoneal injections of LPS (2.5 mg/kg body weight, L2880, Sigma) for 2 consecutive days as positive controls, while other groups received PBS. For EVs interventions, ABX mice were administered 100 µg EVs via oral gavage daily for 5 days, with PBS serving as control. The degradation-resistant GLP-2 analog (Cat Number: 223460-79-5, Creative Peptides) was dissolved in saline and administered subcutaneously at 600 µg/kg immediately after reperfusion, followed by twice-daily injections for 3 consecutive days. Control mice received equivalent volumes of saline. The dosage was determined based on previously published studies [2-4].

Myocardial Ischemia-Reperfusion Model [5]: Mice were anesthetized with isoflurane (induction: 4%, maintenance: 1.5-2%) and mechanically ventilated (positive end-expiratory pressure: 3 cm H2O, respiratory rate: 110 breaths/min, tidal volume: 1.2-1.4 mL). Myocardial ischemia was induced by temporary ligation of the left anterior descending coronary artery using 7-0 suture for 60 minutes, followed by reperfusion. Electrocardiographic monitoring was performed throughout the procedure, and echocardiography confirmed successful model establishment. Sham-operated mice underwent identical procedures without coronary artery ligation or I/R injury.

**FITC-dextran gavage**

Mice were administered 50 mg/100g body weight of FITC-dextran 4000 (cat. no. 46944, Sigma-Aldrich). Four hours after administration, the mice were anesthetized via intraperitoneal injection. Peripheral blood was collected, and serum was separated from the blood. After dilution, the serum samples were detected using spectrofluorometry. The detection conditions were set as follows: excitation wavelength at 485 nm and emission wavelength at 528 nm. For quantitative analysis, a series of serially diluted FITC-dextran standards were used, and the final fluorescence values were normalized against the control group.

**Echocardiography**

Cardiac function was evaluated weekly for 4 consecutive weeks following myocardial ischemia-reperfusion injury using high-resolution transthoracic echocardiography (Visual Sonics, Vevo 3100) under light anesthesia. Two-dimensional imaging was performed in both short-axis and long-axis views to obtain comprehensive cardiac parameters. LV end-systolic diameter (LVID;d), LV end-diastolic diameter (LVID;s), interventricular septal thickness (IVS), and LV posterior wall thickness (end-diastolic and end-systolic) were measured from at least three consecutive cardiac cycles on M-mode tracings. LV fractional shortening (FS %) was determined as [(LVID;d - LVID;s)/LVID;d] ×100. LV ejection fraction (EF) was calculated as: EF(%) = ((LV Vol;d - LV Vol;s)/LV Vol;d)×100. Here, LV Vol;d = ((7.0 / (2.4 + LVID;d)) × LVID;d³) and LV Vol;s = ((7.0 / (2.4 + LVID;s)) × LVID;s³).

**TLR4 Signal Assays**

HEK-Blue™ mTLR4 cells were purchased from Invivogen. When the cells reached the indicated density, a cell suspension was prepared using HEK-Blue Detection medium at a concentration of 200,000–300,000 cells/mL. The cell suspension was added to a 96-well plate, and the corresponding intervention measures were applied simultaneously. The plate was incubated in a 37°C incubator with 5% CO₂ for 9 hours. Finally, the absorbance of each well was measured at a wavelength of 630 nm using a microplate reader.

**Flow cytometry**

Blood samples were collected in heparinized tubes and subjected to red blood cell lysis (00-4300-54, eBioscience). Tissue samples from the left ventricular apex and spleen were harvested in cold FACS buffer (2% FBS, 0.05% NaN3 in PBS) and mechanically dissociated using the gentleMACS™ Dissociator (Miltenyi Biotec) to obtain single-cell suspensions. The suspensions were subsequently filtered through 70 μm cell strainers (BD Biosciences Pharmingen) to remove debris.

For immunophenotyping, cell suspensions were incubated with fluorochrome-conjugated antibodies (1 µL per 10^6 cells) at 4°C for 30 minutes in the dark. The following antibody panel was used: CD45 (PerCP-Cyanine5.5, 45-0451-82, eBioscience), F4/80 (PerCP-Cyanine5.5, 45-4801-82, eBioscience), CD11b (FITC, 11-0112-82, eBioscience), Ly-6C (APC, 17-5932-82, eBioscience; PE, 130-111-916, Miltenyi Biotec), Ly-6G (APC, 17-9668-82, eBioscience). Following staining, cells were washed twice with PBS and resuspended in PBS containing 5% FBS for flow cytometric analysis using the BD Accuri C6 Plus system. Data acquisition and analysis were performed using FlowJo software (version 10, Tree Star, Ashland, OR), with appropriate gating strategies applied to identify specific immune cell populations.

**Flow cytometry of bead-bound EVs**

The experimental procedures were performed according to established protocols with appropriate modifications, as previously described in the literature [6]. Approximately 5 × 10^9^ EVs were resuspended in 100 μL PBS and conjugated with 10 μL aldehyde/sulfate latex beads (Invitrogen, A37304) through 15-minute incubation at room temperature with constant rotation. The mixture was then diluted with 200 μL PBS and allowed to bind overnight at 4°C with continuous rotation. To quench unreacted aldehyde groups, 150 μL of 1 M glycine was added to each sample, followed by 1-hour incubation at room temperature with rotation. The bead-bound EVs were pelleted by centrifugation at 13,000 g for 1.5 minutes and subsequently blocked with 100 μL of 10% BSA for 1 hour at room temperature with rotation. For immunostaining, the blocked bead-EV complexes were incubated with primary antibodies against OmpA (orb422682, Biorbyt) and OmpC (orb686344, Biorbyt) for 1 hour at room temperature with vortexing and rotation. Following three washes with 200 μL of 2% BSA (13,000 g, 1.5 minutes each), the samples were incubated with fluorochrome-conjugated secondary antibodies (goat anti-rabbit IgG(H+L) Alexa Fluor 488, 711-175-152 ,Jackson immunoresearch) in 20 μL of 2% BSA for 1 hour with rotation. After secondary antibody staining, the samples underwent three additional washes with 2% BSA. Finally, the immunostained bead-EV complexes were resuspended in 2% BSA/PBS for flow cytometric analysis using the BD Accuri C6 Plus system. Data acquisition and analysis were performed using FlowJo software (version 10, Tree Star, Ashland, OR), with appropriate gating strategies applied to quantify EV subpopulations.

**Immunofluorescence staining**

Tissue samples were fixed in 4% paraformaldehyde for 2-3 hours at room temperature, followed by PBS washing for 20 minutes. For cryoprotection, samples were immersed in PBS containing 30% sucrose overnight at 4°C and subsequently embedded in Tissue-Tek OCT compound (SAKURA, 4583) for cryosectioning. Coronal sections (8 μm thickness) were prepared using a cryostat and incubated in blocking buffer containing 10% goat serum for 1 hour at room temperature. Primary antibodies diluted in blocking buffer were applied to tissue sections and incubated overnight at 4°C. After three PBS washes, sections were incubated with appropriate secondary antibodies and DAPI (1 μg/mL, Sangon Biotech, E607303-0002) for 2 hours at room temperature. Following three additional PBS washes, sections were mounted and imaged using the THUNDER Imaging System. Image acquisition parameters were kept consistent across all experimental groups to ensure comparability. The following antibodies were used: Anti-claudin-1 (PA1-37464, ThermoFisher, 1:200), Anti-CD68 (ab53444, Abcam, 1:200), Anti-iNOS (ab3523, Abcam, 1:50), Goat anti-rabbit IgG (H+L) Alexa Fluor 488 (112-545-144, Jackson ImmunoResearch, 1:200), Goat anti-rat IgG (H+L) Alexa Fluor 488 (112-545-167, Jackson ImmunoResearch, 1:200), Goat anti-rabbit IgG (H+L) Cy3 (111-165-144, Jackson ImmunoResearch, 1:200).

**Masson trichrome, Sirius red staining, TTC and H&E staining**

Heart tissues were harvested at appropriate time points, fixed in 10% neutral buffered formalin, and embedded in paraffin. Serial sections (5 μm thickness) were prepared for histological analysis. Tissue sections were stained with Masson's trichrome, Sirius red and hematoxylin-eosin (H&E) following standard manufacturer's protocols.

To quantify inflammatory cell infiltration in myocardial tissue, ten random fields within the infarct area of each heart were selected for analysis. The percentage of inflammatory cells was determined by counting the number of inflammatory cell nuclei and normalizing to the total number of nuclei in the same field of view [7].

Intestinal mucosal damage was evaluated using the modified Chiu scoring system [2, 8-10] as follows: Grade 0: Normal mucosal architecture. Grade 1: Development of subepithelial space at villus tips with capillary congestion. Grade 2: Moderate upward pushing of mucosal epithelium by subepithelial space formation. Grade 3: Extensive upward lifting of mucosal epithelium along villi with focal tip denudation. Grade 4: Complete villus denudation reaching the lamina propria with capillary dilatation. Grade 5: Presence of ulceration, lamina propria disintegration, and hemorrhage.

For quantitative assessment of myocardial infarct size, Evans blue/TTC double staining was performed on day 3 post ischemia-reperfusion injury. Briefly, Evans blue (1% in saline) was administered via aortic perfusion to delineate the non-ischemic area (blue staining). Following thorough perfusion, hearts were excised and sectioned into 1-mm thick slices perpendicular to the long axis. Tissue sections were incubated in 1% 2,3,5-triphenyl-2H-tetrazolium chloride (TTC, Sigma, T8877) in PBS at 37°C for 20 minutes, protected from light. The area stained blue by Evans blue indicated the area not at risk, whereas the unstained tissues represented area-at-risk (AAR). AAR but viable tissue was stained red by TTC, while the infracted myocardium was not stained by any dye and appeared more pale than other areas. All quantitative statistics were performed using Image-Pro Plus software.

**ELISA**

Inflammatory factors and LPS were detected by ELISA kits following the manufacturer’s protocols (Mouse TNF-α, EK282HS, Multisciences; Mouse IL-6, EK206, Multisciences; Mouse IL-1β, EK201B, Multisciences; Mouse LPS, CSB-E13066m, Cusabio; Human LPS CSB-E09945h, Cusabio).

**SUPPLEMENTAL REFERENCES**

1. Bittel M, Reichert P, Sarfati I, Dressel A, Leikam S, Uderhardt S, et al. Visualizing transfer of microbial biomolecules by outer membrane vesicles in microbe-host-communication in vivo. *J Extracell Vesicles*. 2021;10:e12159

2. Zhao J, Zhang Q, Cheng W, Dai Q, Wei Z, Guo M, et al. Heart-gut microbiota communication determines the severity of cardiac injury after myocardial ischaemia/reperfusion. *Cardiovasc. Res.* 2023;119:1390-1402

3. Lei Q, Bi J, Wang X, Jiang T, Wu C, Tian F, et al. Glp-2 prevents intestinal mucosal atrophy and improves tissue antioxidant capacity in a mouse model of total parenteral nutrition. *Nutrients*. 2016;8

4. Cheng W, Wang K, Zhao Z, Mao Q, Wang G, Li Q, et al. Exosomes-mediated transfer of mir-125a/b in cell-to-cell communication: A novel mechanism of genetic exchange in the intestinal microenvironment. *Theranostics*. 2020;10:7561-7580

5. Wang J, Tan Y, Dai Y, Hu K, Tan X, Jiang S, et al. Intranasal delivery of endothelial cell-derived extracellular vesicles with supramolecular gel attenuates myocardial ischemia-reperfusion injury. *Int J Nanomedicine*. 2023;18:5495-5510

6. Kugeratski FG, Hodge K, Lilla S, McAndrews KM, Zhou X, Hwang RF, et al. Quantitative proteomics identifies the core proteome of exosomes with syntenin-1 as the highest abundant protein and a putative universal biomarker. *Nat. Cell Biol.* 2021;23:631-641

7. Zhao J, Li X, Hu J, Chen F, Qiao S, Sun X, et al. Mesenchymal stromal cell-derived exosomes attenuate myocardial ischaemia-reperfusion injury through mir-182-regulated macrophage polarization. *Cardiovasc. Res.* 2019;115:1205-1216

8. Zhang J, Yu WQ, Wei T, Zhang C, Wen L, Chen Q, et al. Effects of short-peptide-based enteral nutrition on the intestinal microcirculation and mucosal barrier in mice with severe acute pancreatitis. *Mol Nutr Food Res*. 2020;64:e1901191

9. Chiu CJ, McArdle AH, Brown R, Scott HJ, Gurd FN. Intestinal mucosal lesion in low-flow states. I. A morphological, hemodynamic, and metabolic reappraisal. *Arch. Surg.* 1970;101:478-483

10. Wenying S, Jing H, Ying L, Hui D. The role of TLR4/MyD88/NF-kappaB in the protective effect of ulinastatin on the intestinal mucosal barrier in mice with sepsis. *BMC Anesthesiol*, 2023;23, 414. 10.1186/s12871-023-02374-9
